# A systematic review and meta-analysis of the effectiveness of food safety education interventions for consumers in developed countries

**DOI:** 10.1186/s12889-015-2171-x

**Published:** 2015-08-26

**Authors:** Ian Young, Lisa Waddell, Shannon Harding, Judy Greig, Mariola Mascarenhas, Bhairavi Sivaramalingam, Mai T. Pham, Andrew Papadopoulos

**Affiliations:** Laboratory for Foodborne Zoonoses, Public Health Agency of Canada, 160 Research Lane, Suite 206, Guelph, ON N1G 5B2 Canada; Department of Population Medicine, University of Guelph, 50 Stone Road, Guelph, ON N1G 2W1 Canada

## Abstract

**Background:**

Foodborne illness has a large public health and economic burden worldwide, and many cases are associated with food handled and prepared at home. Educational interventions are necessary to improve consumer food safety practices and reduce the associated burden of foodborne illness.

**Methods:**

We conducted a systematic review and targeted meta-analyses to investigate the effectiveness of food safety education interventions for consumers. Relevant articles were identified through a preliminary scoping review that included: a comprehensive search in 10 bibliographic databases with verification; relevance screening of abstracts; and extraction of article characteristics. Experimental studies conducted in developed countries were prioritized for risk-of-bias assessment and data extraction. Meta-analysis was conducted on data subgroups stratified by key study design-intervention-population-outcome categories and subgroups were assessed for their quality of evidence. Meta-regression was conducted where appropriate to identify possible sources of between-trial heterogeneity.

**Results:**

We identified 79 relevant studies: 17 randomized controlled trials (RCTs); 12 non-randomized controlled trials (NRTs); and 50 uncontrolled before-and-after studies. Several studies did not provide sufficient details on key design features (e.g. blinding), with some high risk-of-bias ratings due to incomplete outcome data and selective reporting. We identified a moderate to high confidence in results from two large RCTs investigating community- and school-based educational training interventions on behaviour outcomes in children and youth (median standardized mean difference [SMD] = 0.20, range: 0.05, 0.35); in two small RCTs evaluating video and written instructional messaging on behavioural intentions in adults (SMD = 0.36, 95 % confidence interval [CI]: 0.02, 0.69); and in two NRT studies for university-based education on attitudes of students and staff (SMD = 0.26, 95 % CI: 0.10, 0.43). Uncontrolled before-and-after study outcomes were very heterogeneous and we have little confidence that the meta-analysis results reflect the true effect. Some variation in outcomes was explained in meta-regression models, including a dose effect for behaviour outcomes in RCTs.

**Conclusions:**

In controlled trials, food safety education interventions showed significant effects in some contexts; however, many outcomes were very heterogeneous and do not provide a strong quality of evidence to support decision-making. Future research in this area is needed using more robust experimental designs to build on interventions shown to be effective in uncontrolled before-and-after studies.

**Electronic supplementary material:**

The online version of this article (doi:10.1186/s12889-015-2171-x) contains supplementary material, which is available to authorized users.

## Background

Foodborne illness has a large public health and economic burden worldwide. For example, an estimated 48 million cases of foodborne illness occur each year in the United States (US), causing approximately 128,000 hospitalizations and 3000 deaths [1, 2]. In addition, 14 major foodborne pathogens are estimated to cause US$14.0 billion and a loss of 61,000 quality-adjusted life years annually [3]. In Canada, approximately 4 million cases of foodborne illness occur each year [4], with acute gastroenteritis estimated to cost $3.7 billion annually [5].

Reliable data on the burden of foodborne illness due to consumer mishandling of food prepared and consumed in domestic households is not routinely and consistently collected and reported in many countries. However, previous research suggests that most sporadic cases of foodborne illness, which are often underreported and underdiagnosed, are more frequently associated with food consumed at home than other settings [6–8], and across Europe reported outbreaks of foodborne illness are largely associated with domestic household kitchens [9]. Many consumers tend to expect the foods they purchase to be safe and believe that there is a low risk of becoming ill from food prepared and consumed in their home [8, 10, 11]. In addition, previous surveys of food safety behaviours among consumers in the US, Canada, and the United Kingdom have found that many consumers do not follow key safe food handling recommendations [8, 11–13]. These studies, as well as government outbreak reports and food safety policy documents [14–17], have identified a need for enhanced food safety education for consumers in targeted areas.

Educational interventions for consumers are necessary to increase their knowledge and awareness about food safety, to change their food handling and preparation behaviours, and ultimately, to decrease the incidence and burden of foodborne illness due to food prepared and handled at home [18–20]. There is a need to update and expand upon previous systematic reviews conducted in this area, which are significantly outdated [21, 22] or had restricted inclusion criteria for the interventions and study designs considered [19]. Therefore, we conducted a comprehensive scoping and systematic review to synthesize the effectiveness of all types of food safety educational interventions for consumers. We report here on the systematic review component of this project; the scoping review results are summarized and reported in a separate publication [23]. This review was reported in accordance with the PRISMA guidelines [24] (see checklist in Additional file 1).

## Methods

### Review team, question, scope, and eligibility criteria

The review followed a protocol that was developed a priori and is available from the corresponding author upon request; methods followed standard guidelines for scoping and systematic reviews [25, 26]. The core review team consisted of seven individuals with complementary topic (i.e. food safety education) and methodological (i.e. knowledge synthesis) expertise. In addition, we engaged six knowledge-users in the review through an expert advisory committee [27]. The committee was engaged using an e-mailed questionnaire once before the review proceeded to provide input on the review scope, inclusion criteria, and search strategy, and again after completion of the scoping review stage to provide input on the article characterization results and the prioritization of articles for systematic review (risk-of-bias assessment and data extraction) and meta-analysis.

The key review question was “What is the effectiveness of targeted educational interventions to improve consumer food safety knowledge, attitudes, and behaviours?” Interventions of interest were categorized into two broad categories: 1) training workshops, courses, and curricula in school, academic, and community settings; and 2) social marketing campaigns and other types of educational messaging materials, such as print media (e.g. exposure to brochures, website information, food product label information) and audio-video media (e.g. radio or TV ads). The review scope included primary research published in English, French, or Spanish, with no publication date restrictions, in any of the following document formats: peer-reviewed journal articles, research reports, dissertations, and conference abstracts or papers. Interventions that did not have an explicit food safety component were excluded (e.g. generic hand-washing not in a food handling context). Consumers were defined as those who prepare or handle food for consumption at home, including volunteer food handlers for special events (e.g. potlucks). We also included studies targeted at educators of consumers (e.g. train-the-trainer studies). Studies targeted at food handlers employed in the food service industry were excluded [28].

### Search strategy and scoping review methods

A comprehensive and pre-tested search strategy was implemented on May 20, 2014, in 10 bibliographic databases: Scopus, PubMed, Agricola, CAB Abstracts, Food Safety and Technology Abstracts, PsycINFO, Educational Resources Information Center (ERIC), Cumulative Index to Nursing and Allied Health Literature (CINAHL), ProQuest Public Health, and ProQuest Dissertations and Theses. The search algorithm comprised a targeted combination of food safety-related terms (e.g. food safety, food hygiene), population-setting terms (e.g. consumer, adults, home), intervention terms (e.g. program, course, campaign), and outcome terms (e.g. behaviour, knowledge, attitudes). The search was verified by hand-searching two journals (Environmental Health Review and the Journal of Nutrition Education and Behavior “Great Educational Materials” Collection), reviewing the websites of 24 relevant organizations, and reviewing the reference lists of 15 review articles and 15 relevant primary research articles.

The titles and abstracts of identified citations were screened for relevance to the review question using a pre-specified and pre-tested form. The form was also used to identify review articles to be used for search verification. Potentially relevant citations were then procured as full articles, confirmed for relevance, and characterized using a pre-specified and pre-tested form consisting of 29 questions about the article type, study design, data collection methods, and details of the interventions, populations, and outcomes investigated. Full details on the search strategy, including database-specific algorithms, and a copy of the screening and characterization forms are reported in Additional files 2 and 3.

### Risk-of-bias assessment and data extraction

In consultation with the expert advisory committee, we decided to limit further analysis to experimental studies (randomized and non-randomized controlled trials and uncontrolled before-and-after studies) conducted in North America, Europe, Australia, and New Zealand. The rationale for this decision was that these studies were deemed to provide the most relevant evidence to our main stakeholders (Canadian food safety decision-makers and practitioners). All relevant studies meeting these criteria were assessed for their risk of bias at the outcome-level and relevant outcomes were extracted using two pre-specified forms applied in sequence (Additional file 3). The risk-of-bias form contained four initial screening questions to confirm eligibility followed by up to 12 risk-of-bias criteria questions depending on study design, including an overall risk-of-bias rating for each main outcome. Each criterion was rated as low, unclear, or high risk. The risk-of-bias criteria were adapted from existing tools for randomized and non-randomized experimental studies [26, 29, 30]. Outcome data and quantitative results were then extracted from each study for each intervention-population-outcome combination reported.

### Review management

Citations identified in the search were uploaded to RefWorks (Thomson ResearchSoft, Philadelphia, PA) and duplicates were removed manually. Citations were imported into the web-based systematic review software DistillerSR (Evidence Partners, Ottawa, ON, Canada), which was used to conduct each stage of the scoping and systematic review (from relevance screening to data extraction). Results were exported as Microsoft Excel spreadsheets for formatting and analysis (Excel 2010, Microsoft Corporation, Redmond, WA).

The relevance screening and article characterization forms were pre-tested by nine reviewers on 50 and 10 purposively-selected abstracts and articles, respectively. Reviewing proceeded when kappa scores for inclusion/exclusion agreement between reviewers was >0.8. The risk-of-bias and data extraction forms were pre-tested by three reviewers (I.Y., L.W., and S.H.) on six articles. In all cases, the pre-test results were discussed among reviewers and forms were revised and clarified as needed. Nine reviewers conducted the scoping review stages (relevance screening and article characterization) and two reviewers conducted risk-of-bias assessment and data extraction (I.Y. and S.H.). For all stages, two independent reviewers assessed each citation or article. Disagreements between reviewers were resolved by consensus, and when necessary, by judgement of a third reviewer.

### Meta-analysis

Relevant studies were stratified into subgroups for meta-analysis [26, 31]. Firstly, studies were stratified into three main groups of study designs: 1) randomized controlled trials (RCTs); 2) non-randomized controlled trials (NRTs); and 3) uncontrolled before-and-after studies. Secondly, data were stratified into the two intervention categories of interest (training workshops/courses and social marketing campaigns/other messaging). Data were then stratified by target population into three main categories: 1) children and youth (<18 years old); 2) adults (18 and older); and 3) educators of consumers. Within each of these subgroups, three main outcome types were considered: 1) knowledge; 2) attitudes; and 3) behaviours. Two additional theoretical construct outcomes investigated in a smaller number of studies were also assessed: 4) behavioural intentions; and 5) stages of change [32, 33]. Separate meta-analyses were then conducted in each data subgroup for dichotomous and continuous outcome measures when sufficiently reported data were available from ≥2 studies. Dichotomous analyses were conducted using the relative risk (RR) metric and continuous data were analyzed using the standardized mean difference (SMD; Hedge’s g), which accounts for the variable and non-standardized outcome scales reported across studies [26, 31]. All models were conducted using the DerSimonian and Laird method for random-effects [34]. The unit of analysis was individual trials (intervention-population-outcome combinations) reported within studies.

Many studies with continuous outcomes did not report required standard deviations to allow for meta-analysis; in these cases, other reported summary statistics (e.g. confidence intervals, standard errors, t values, *P* values, F values) were used to approximate the missing values using the formulas described in Higgins and Green (2011) [26] and implemented in CMA software (Comprehensive Meta-Analysis Version 2, Biostat, Inc., Englewood, NJ). For meta-analyses of RCTs and NRTs, some studies reported differences in changes from baseline (pre-to-post tests) between study groups; these were combined in the same analysis as studies reporting differences in final outcome measures [31, 35]. When these studies did not report the standard deviation of the mean change or other summary statistics as described above necessary to approximate this value, only final outcome measures were used in analysis if baseline measurements were similar. When baseline measurements differed, best available estimates of the pre-post correlation value were imputed from previous studies in the literature that examined similar outcomes in similar populations [26, 31]. Specifically, a pre-post correlation of 0.81 was used for knowledge and attitude outcomes [36] and a value of 0.83 was used for behaviour outcomes [37] (Additional file 4). The same imputations were conducted for all meta-analyses of SMD measures in uncontrolled before-and-after studies, as none of these studies reported pre-post correlation values necessary to conduct an appropriate paired analysis. Sensitivity analyses were conducted in each case by comparing to pre-post correlations of 0.2 and 0.9 [26, 31, 38]. Similarly, none of the uncontrolled before-and-after studies measuring dichotomous outcomes reported data in a matched format; therefore, these outcomes were analyzed as unmatched data, which has been shown to be similar and easier to interpret than matched analyses [39]. Finally, some studies reported the number of participants in >2 ordinal categories (e.g. always, usually, sometimes, never); for ease of analysis and interpretation, these outcomes were dichotomized into the most logical categories based on their comparability to other dichotomous data available in the same data subset.

Some studies reported results for multiple outcomes measuring the same construct (e.g. knowledge scores) in the same group of participants. To avoid counting the same participants more than once in the same meta-analysis, we computed a combined measure of effect for each outcome in these studies [31]. The combined effect was taken as the mean of the individual measures, while the variance was calculated using the following formula [31]:

1 $$ {V}_{\overline{Y}}={\left(\frac{1}{m}\right)}^2var\left({\displaystyle {\sum}_{j=1}^m{Y}_i}\right)={\left(\frac{1}{m}\right)}^2\left({\displaystyle {\sum}_{j=1}^m{V}_i}+{\displaystyle {\sum}_{j\ne k}}\left({r}_{jk}\sqrt{V_j}\sqrt{V_k}\right)\right), $$

where *m* indicates the number of outcomes being combined, *V* indicates the variance of the *j*th and *k*th outcomes being combined, and *r* refers to the correlation between each two constructs being combined. Unfortunately, a measure of the correlation (*r*) between each pair of constructs was only reported for one of the study outcomes combined in this manner [40]. For all other studies, we imputed plausible correlation values taken from averages reported in other relevant studies in the literature that tested or evaluated food safety knowledge, attitude, or behaviour questionnaires in similar populations and contexts [36, 40–42]. Specifically, we used average correlation values of 0.36, 0.47, and 0.62 for knowledge, attitude, and behaviour outcomes, respectively, and conducted a sensitivity analysis in each case by comparing to values of 0.2 and 0.8 to identify potential impacts on the outcomes using a range of possible values [31] (Additional file 4).

In studies that compared more than one intervention and/or control group, one of the following decisions was made on a case-by-case basis depending on the nature of the groups being compared and their relevance to the review question: 1) groups were combined into a single pair-wise comparison using the formula described in Higgins and Green (2011) [26]; or 2) the control group was split into two or more groups with a smaller sample size. A table outlining the selected approach and decision in each of these cases is shown in the supplementary materials (Additional file 5). For studies that reported outcome measurements for multiple time points (e.g. pre, post, and follow-up), we used the pre-to-post measure in the meta-analysis calculation as this was most comparable to what other studies reported across all subgroups [43]. Sensitivity analyses were conducted in these cases by repeating the analysis with the pre-to-follow-up measures to explore the impact of a longer follow-up on the intervention effect.

Heterogeneity in all meta-analyses was measured using *I*^2^, which indicates the proportion of variation in effect estimates across trials that is due to heterogeneity rather than sampling error [44]. Heterogeneity was considered high and average estimates of effect were not shown when *I*^2^ > 60 % [26, 44]. In these cases, a median and range of effect estimates from individual trials in the meta-analysis subgroup was shown instead, as presenting pooled meta-analysis estimates in the presence of so much variation can be misleading [45]. Meta-analysis effect estimates were considered significant if the 95 % confidence intervals (CI) excluded the null. Begg’s adjusted rank correlation and Egger’s regression tests were used to test for possible publication bias on meta-analysis data subsets with ≥10 trials and when heterogeneity was not significant [46]. For these tests, *P* < 0.05 was considered significant. All meta-analyses were conducted using CMA software.

### Meta-regression

Meta-regression was conducted on meta-analysis data subsets with *I*^2^ > 25 % and ≥10 trials to explore possible sources of heterogeneity in the effect estimates across trials [47]. To increase power of these analyses, data were not stratified by intervention type or population subgroup; instead, these two variables were evaluated as predictors of heterogeneity in outcomes across trials. In addition, the following 15 pre-specified variables were evaluated as potential predictors in meta-regression models: publication year (continuous); document type (journal vs. other); study region (North America vs. other); food safety-specific intervention vs. inclusion of other content (e.g. nutrition) (yes vs. no); intervention development informed by a theory of behaviour change (yes vs. no) or formative research (yes vs. no); target population engaged in intervention development, implementation, and/or evaluation (yes vs. no); intervention included a digital/web-based (yes vs. no) or audio-visual (yes vs. no) component; intervention targeted high-risk (yes vs. no) or low socio-economic status (yes vs. no) populations; overall risk-of-bias rating (low vs. unclear/high); whether any outcomes were insufficiently reported to allow for meta-analysis (yes vs. no); length of participant follow-up (within two weeks post intervention/not reported vs. longer); and intervention dose (>1 vs. only one exposure/not reported). A dose effect of >1 represented interventions with multiple training sessions or lessons and messaging interventions with more than one medium or exposure type (i.e. multifaceted interventions). High-risk populations referred to infants, the elderly, the immuno-compromised, caregivers of these populations, and pregnant women. Two additional variables were also evaluated in RCT and NRT sub-groups: 1) whether the intervention was compared to a positive control group (e.g. standard training) vs. a negative control; and 2) whether the trial was analyzed using unpaired or paired (change from baseline) data.

Given the limited number of trials in each meta-analysis subset, all predictors except publication year were modelled as dichotomous variables. In addition, only univariable meta-regression models were evaluated when the number of trials was 10–19. When the number of trials was ≥20, predictors were initially screened in univariable models and then added in multivariable models using a forward-selection process, up to a maximum of one predictor per 10 trials. Predictors were considered significant if 95 % CIs excluded the null. For each data subgroup, Spearman rank correlations were used to evaluate collinearity between variables prior to conducting meta-regression; if evidence of collinearity was identified (ρ ≥ 0.8), only one of the correlated variables was modelled based on its relevance. Meta-regression was conducted using Stata 13 (StataCorp, College Station, TX).

### Quality-of-evidence assessment

Each meta-analysis data subgroup was assessed for its overall quality-of-evidence using a modified version of the Cochrane Collaboration’s Grades of Recommendation, Assessment, Development and Evaluation (GRADE) approach [26, 48]. Datasets started with 2–4 points to reflect inherent differences in strength of evidence by study design: RCTs started with four points, NRTs with three, and uncontrolled before-and-after studies with two. Points were deducted or added based on the five downgrading and three upgrading criteria described in Table 1. The final GRADE rating corresponded to the remaining number of points: one = very low (the true effect is likely to be substantially different from the measured estimate); two = low (the true effect may be substantially different from the measured estimate); three = moderate (the true effect is likely to be close to the measured estimate, but there is a possibility that it is substantially different); four = high (we have strong confidence that the true effect lies close to that of the measured estimate).

**Table 1:** Modified GRADE approach for evaluating the quality of evidence of meta-analysis data subgroups

Criteria	GRADE Points	Explanation
Downgrading criteria		
1. Individual study risk-of-bias rating and reporting limitations		One point deducted for each criterion where conditions are met. Sensitivity analysis considered to have appreciable impact if range of values changed estimates by >20 % or changed significance of overall effect.
a) >50 % of trials had an unclear or high overall risk-of-bias rating	a) = −1 b) = −1	
b) Key assumptions/imputed values due to reporting limitations had appreciable impact on results in sensitivity analysis		
2. Inconsistency of direction and heterogeneity of findings among studies		Heterogeneity in the results was measured by *I* ^2^. Consistency considered when the individual study estimates in the meta-analysis all show the same direction of effect.
a) Consistent direction of effect, but significant heterogeneity	a) = −1 b) = −2	
b) Inconsistent direction of effect and significant heterogeneity		
3. Imprecision of effect estimates	a) = −1	Power calculations conducted assuming α = 0.05 and *β* = 0.2. For continuous outcomes, power calculated for a difference in means of 0.5 using a range of representative standard deviations from the meta-analysis subgroup. For dichotomous outcomes, power calculated using a relative risk reduction of 30 % and median control group risk from the meta-analysis subgroup.
a) The total number of participants in the meta-analysis subgroup is less than that required by a conventional sample size calculation for a single adequately powered controlled trial		
4. Indirectness of individual study parameter as representative of target parameter		Indirectness indicates studies did not directly measure the target parameter of interest to the review question (e.g. food safety outcomes only reported as part of a combined score/scale with other constructs such as nutrition).
a) >50 % of trials indirectly measure the intervention, population, comparison, or outcome	a) = −1 b) = −2	
a) >50 % of trials measure two or more of the above parameters indirectly		
5. Publication bias	a) = −1	This criterion can only be evaluated if publication bias assessment is possible based on the nature of the data (i.e. ≥10 studies, non-significant heterogeneity, and at least some of the studies have significant results).
a) Detected or suspected in data subset		
Upgrading criteria		
1. Large magnitude of effect	a) = +1	Large effect considered at least a 2-fold reduction in risk.
a) Large effect in the absence of plausible confounders and major threats to validity		
2. Results may have been underestimated due to the study design (e.g. population sampled)	a) = +1	E.g. intervention was tested only on individuals with prior food safety knowledge/training, and it is likely that a stronger effect would have been found if the intervention was tested in the general consumer population.
a) Criterion present		
3. Dose-response gradient	a) = +1	Meta-regression dose variable represents >1 training course/session or multifaceted messaging interventions vs. a single course/session or provision of messaging materials through a single medium or exposure type.
a) >50 % of trials identified a dose-response relationship OR dose identified as significant in meta-regression.		

## Results

### Review flow chart and risk-of-bias results

**Fig. 1:**
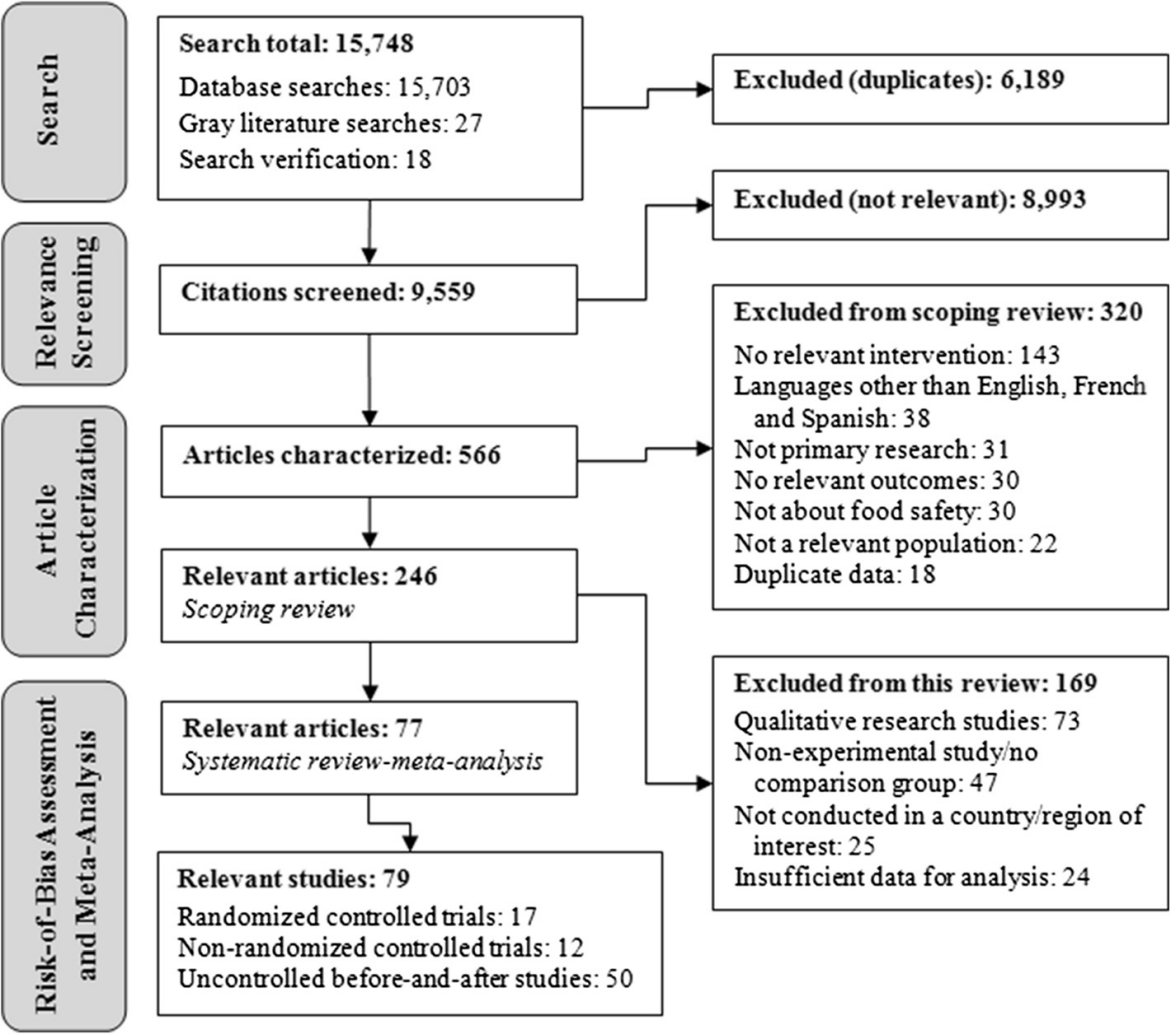
Scoping and systematic review flow-chart. Languages excluded during article characterization included: Chinese (*n* = 11), Korean (8), Portuguese (5), Japanese (5), Italian (2), German (2), Turkish (2), Polish (1), Lithuanian (1), and Hebrew (1). Note: Two of the 77 relevant articles reported more than one study design

A flow chart of the scoping and systematic review process is shown in Fig. 1. From 246 articles considered relevant in the scoping review, 77 met the inclusion criteria for this systematic review (Fig. 1). A citation list of these 77 articles is reported in Additional file 6. The 77 articles reported on 79 unique study designs, including 17 RCTs, 12 NRTs, and 50 uncontrolled before-and-after studies. Most studies (82 %, *n* = 65) were conducted in the United States, compared to 14 % (*n* = 11) in Europe, 3 % (*n* = 2) in Australia, and 1 % (*n* = 1) in Canada. A summary table of the key population, intervention, comparison, and outcome characteristics of each study is shown in Additional file 7. Full descriptive results for the scoping review stages (relevance screening and article characterization) are reported in a separate publication [23].

The risk-of-bias ratings are shown stratified by study design in Table 2, with detailed results of the within-study assessments shown in Additional file 8. Many RCTs did not provide sufficient details on their methods of random sequence generation and allocation concealment. Blinding criteria was also unclear for many studies across all designs (Table 2). Some unclear and high risk ratings were noted due to incomplete outcome data and selective reporting (Table 2). Many uncontrolled before-and-after studies (17/50) also did not provide details on the validity and reliability of outcome measurement instruments, leading to an unclear rating for that criterion.

**Table 2:** Risk-of-bias rating summary for studies investigating the effectiveness of food safety education interventions for consumers

	No. of studies low/unclear/high risk		
Criteria	RCT (*n* = 17)	NRT (*n* = 12)	UBA (*n* = 50)
Random sequence generation	6/11/0	N/A^a^	N/A^a^
Allocation concealment	11/6/0	9/3/0	N/A^a^
Similarity of baseline outcomes	13/4/0	8/4/0	N/A^a^
Similarity of baseline characteristics	10/6/1	8/4/0	N/A^a^
Blinding of participants and personnel	6/11/0	4/8/0	N/A^a^
Blinding of outcome assessment:			
Knowledge	13/4/0	8/4/0	N/A^a^
Other outcomes	8/9/0	4/8/0	N/A^a^
Blinding of participants to the research question/purpose	N/A^a^	N/A^a^	16/30/4
Independence of intervention effect from confounding bias	15/2/0	11/1/0	48/1/1
Valid/reliable outcome measurement	14/3/0	10/2/0	33/17/0
Incomplete outcome data:			
Knowledge	12/2/3	8/3/1	31/19/0
Other outcomes	12/2/3	8/3/1	30/20/0
Selective reporting	15/0/2	9/2/1	40/9/1
Other	14/3/0	12/0/0	48/2/0
Overall risk-of-bias:			
Knowledge	8/6/3	7/4/1	34/15/1
Other outcomes	8/6/3	7/4/1	33/16/1

*RCT* randomized controlled trials, *NRT* non-randomized controlled trials, *UBA* uncontrolled before-and-after studies

^a^N/A = These criteria not assessed for these study designs

### Meta-analysis results

The meta-analysis results for RCTs and NRTs are shown in Table 3. All RCT meta-analyses were significantly heterogeneous except for the effect of messaging materials (instructional video and written messages) on behavioural intentions in adults in two small studies, which showed a positive intervention effect (SMD = 0.36, 95 % CI: 0.02, 0.69; ‘moderate’ GRADE rating). All other outcomes showed positive median effects across trials (Table 3). The effect of community- and school-based educational training interventions on behaviour outcomes in children and youth received the only ‘high’ GRADE rating. Other behaviour, knowledge, and attitude outcomes received ‘low’ and ‘very low’ GRADE ratings. For meta-analyses of NRTs, educational training and course interventions had a positive average estimate of effect on attitudes (SMD = 0.26, 95 % CI: 0.10, 0.43; ‘moderate’ GRADE rating) and behaviours (SMD = 0.37, 95 % CI: 0.08, 0.66; ‘low’ GRADE rating) in adults. Both categories of interventions showed heterogeneous but positive median effects across trials for other outcomes, with ‘low’ and ‘very low’ GRADE ratings (Table 3).

**Table 3:** Random-effects meta-analysis results of randomized and non-randomized controlled trials

Meta-analysis sub-group^a^	No. participants/trials/studies	No. (%) trials with combined outcome measures^b^	Effect measure	Effect estimate average (95 % CI)^M^/median (range)^R,c^	*I* ^2^	GRADE^d^
RCT						
Educational training/courses						
Adults-Behaviours^e^	709/4/4	2 (50)	SMD	0.68 (−0.06, 1.41)^R^	94 %	Low
Adults-Knowledge	596/3/3	0 (0)	SMD	0.87 (−0.05, 1.29)^R^	93 %	Very low
Children/youth-Behaviours	6379/2/2	1 (50)	SMD	0.20 (0.05, 0.35)^R^	96 %	High
Media campaigns/other messaging						
Adults-Behavioural intentions	117/2/2	1 (50)	SMD	0.36 (0.02, 0.69)^M^	0 %	Moderate
Adults-Behaviours	686/4/4	1 (25)	SMD	0.24 (−0.17, 1.03)^R^	85 %	Low
Adults-Knowledge	528/3/3	0 (0)	SMD	0.42 (0.03, 0.92)^R^	82 %	Low
Adults-Attitudes	4914/8/8	4 (50)	SMD	0.34 (0.05, 0.76)^R^	94 %	Low
NRT						
Educational training/courses						
Adults-Behaviours^e^	1099/4/2	0 (0)	SMD	0.37 (0.08, 0.66)^M^	58 %	Low
Adults-Knowledge	1356/5/3	0 (0)	SMD	0.44 (0.12, 1.14)^R^	82 %	Low
Adults-Attitudes	778/4/2	1 (25)	SMD	0.26 (0.10, 0.43)^M^	0 %	Moderate
Children/youth-Behaviours	329/3/2	0 (0)	SMD	0.33 (0.17, 0.90)^R^	64 %	Very low
Children/youth-Knowledge	339/3/2	0 (0)	SMD	0.24 (0.14, 0.73)^R^	75 %	Very low
Media campaigns/other messaging						
Adults-Behaviours	1118/2/2	0 (0)	RR	2.31 (1.30, 3.33)^R^	90 %	Low
Adults-Attitudes	1442/3/3	1 (33)	RR	1.75 (1.01, 2.85)^R^	95 %	Very low

*RCT* randomized controlled trials, *NRT* non–randomized controlled trials, *SMD* standardized mean difference (Hedges’ *g*), *RR* relative risk, *CI* confidence interval

^a^Subgroups divided by study design, intervention type, target population, and outcome type

^b^Refers to studies that reported multiple measures of the same construct in the same individuals, which were combined *post hoc* into one overall measure

^c^Superscript ^M^ indicates that an average estimate of effect and 95 % CI is provided because heterogeneity was low to moderate (*I* ^2^ = 0–60 %). Superscript ^R^ indicates that the median and range of study effect sizes is provided because heterogeneity was high (*I* ^2^ > 60 %)

^d^Explanation of the GRADE ratings:

Very low = the true effect is likely to be substantially different from the measured estimate

Low = the true effect may be substantially different from the measured estimate

Moderate = the true effect is likely to be close to the measured estimate, but there is a possibility that it is substantially different

High = strong confidence that the true effect lies close to that of the measured estimate

^e^One trial/study in each of these analyses used an imputed pre-post correlation value of 0.83 from Kendall et al. (2004) [37]. In both cases, sensitivity analyses indicated that the selection of the imputed value had an appreciable impact on the meta-analysis results (Additional file 12), leading to a downgrading of these findings in the GRADE assessment (Additional file 10)

The meta-analysis results for uncontrolled before-and-after studies are shown in Table 4. All analyses were significantly heterogeneous, except for the effect of educational training and course interventions on improving the behaviours of educators of consumers in two small studies (SMD = 0.44, 95 % CI: 0.33, 0.54). All other intervention, population, and outcome combinations showed positive median effects across trials (Table 4); however, due to risk of bias, heterogeneity, and inconsistencies all meta-analyses of uncontrolled before-and-after studies received a ‘very low’ GRADE rating. It was not possible to assess publication bias statistically in any meta-analysis subgroup. Forest plots of each meta-analysis are shown in Additional file 9 and the detailed GRADE assessments for each subgroup are shown in Additional file 10.

**Table 4:** Random-effects meta-analysis results of uncontrolled before-and-after studies

Meta-analysis sub-group^a^	No. participants/trials/studies^b^	No. (%) trials with combined outcome measures^c^	Effect measure^d^	Effect estimate average (95 % CI)^M^/median (range)^R,e^	*I* ^2^
Educational training/courses					
Educators-Behaviours	85/2/2	1 (50)	SMD	0.44 (0.33, 0.54)^M^	0 %
Educators-Knowledge	47/3/3	2 (67)	RR	2.86 (1.31, 5.63)^R^	79 %
Educators-Attitudes	33/2/2	0 (0)	RR	1.34 (1.06, 1.63)^R^	73 %
Adults-Behaviours	11,764/17/16	8 (47)	SMD	0.28 (0.11, 1.49)^R^	100 %
Adults-Behaviours	3049/10/10	7 (70)	RR	1.26 (0.95, 2.66)^R^	97 %
Adults-Knowledge^f^	1018/8/7	0 (0)	SMD	0.61 (0.01, 1.04)^R^	95 %
Adults-Knowledge	4239/6/6	1 (17)	RR	1.92 (1.18, 3.10)^R^	99 %
Adults-Attitudes^f^	1332/7/7	3 (43)	SMD	0.43 (0.05, 0.95)^R^	99 %
Adults-Attitudes	876/4/4	1 (25)	RR	1.09 (1.04, 1.33)^R^	86 %
Children/youth-Behaviours	401/3/3	0 (0)	SMD	0.31 (0.14, 1.32)^R^	99 %
Children/youth-Behaviours	226/2/2	1 (50)	RR	5.37 (1.04, 9.69)^R^	94 %
Children/youth-Knowledge	1028/6/6	1 (17)	SMD	1.06 (0.09, 4.02)^R^	100 %
Children/youth-Knowledge	1719/6/6	4 (67)	RR	1.77 (1.11, 5.04)^R^	98 %
Children/youth-Attitudes	294/3/3	0 (0)	SMD	0.31 (0.10, 1.32)^R^	99 %
Media campaigns/other messaging					
Adults-Behaviours	2430/7/6	5 (71)	RR	1.35 (0.90, 2.35)^R^	93 %
Adults-Knowledge	1129/7/7	7 (100)	RR	1.58 (1.07, 1.87)^R^	96 %
Adults-Attitudes^f^	1002/3/3	1 (33)	SMD	0.43 (0.13, 0.81)^R^	99 %
Adults-Attitudes	2420/6/5	2 (33)	RR	1.10 (1.02, 1.23)^R^	85 %
Adults-Stages of change^g^	1193/3/2	0 (0)	RR	1.09 (1.07, 1.81)^R^	70 %

*SMD* standardized mean difference (Hedges’ *g*), *RR* relative risk, *CI* confidence interval

^a^Subgroups divided by intervention type, target population, and outcome type. Note that all outcomes in this table had a GRADE rating of very low (the true effect is likely to be substantially different from the measured estimate)

^b^For trials with a RR outcome, the number of participants in this column refers to the number in the post intervention group

^c^Refers to studies that reported multiple measures of the same construct in the same individuals, which were combined *post hoc* into one overall measure

^d^Note that all trials in SMD analyses used imputed values for pre-post correlations of 0.81 for knowledge and attitude outcomes from Medeiros et al. (2004) [36] or 0.83 for behaviour outcomes from Kendall et al. (2004) [37]

^e^Superscript ^M^ indicates that an average estimate of effect and 95 % CI is provided because heterogeneity was low to moderate (*I* ^2^ = 0–60 %). Superscript ^R^ indicates that the median and range of study effect sizes is provided because heterogeneity was high (*I* ^2^ > 60 %)

^f^Sensitivity analyses for these outcomes revealed that the selection of the imputed correlation value had an appreciable impact on the meta-analysis results (Additional file 12), leading to a downgrading of these findings in the GRADE assessment (Additional file 10)

^g^RR for this outcome refers to the impact of the intervention to change participants’ stage from contemplation/pre-contemplation/preparation to action/maintenance [32]

### Meta-regression results

Meta-regression was possible for seven data subgroups: behaviour outcomes in RCTs with the SMD measure, and knowledge, behaviour, and attitude outcomes reported in uncontrolled before-and-after studies for both RR and SMD measures. Significant predictors of between-trial variation were identified for three of these models (Table 5). For the RCT-behaviour outcome, studies that delivered more than one training session or provided messaging materials through more than one medium or exposure type (i.e. multifaceted interventions) found a higher average intervention effect (SMD = 0.68) compared to studies that included only one training session or provided messaging materials through only one medium or exposure (Table 5). For dichotomous knowledge outcomes, uncontrolled before-and-after studies that were published in sources other than journals articles (i.e. theses and reports) reported an average estimate of intervention effect that was 2.01 times more effective than studies published in journal articles (Table 5). For dichotomous behaviour outcomes, uncontrolled before-and-after studies that reported the target population was engaged in the intervention development, implementation, and/or evaluation reported an average estimate of intervention effect that was 1.47 times more effective than studies that did not engage their target population (Table 5).

**Table 5:** Meta-regression results of the impact of selected study-level variables on the meta-analysis estimates

Meta-analysis sub-group/predictor	No. trials/studies	Average estimate of effect (95 % CI)	Adjusted *R* ^2a^
RCT-behaviour-SMD	10/10		
Dose (>1 vs. 1 exposure)		0.68 (0.03, 1.33)	44.6 %
UBA-knowledge-RR	22/20		
Document type (other vs. journal)		2.01 (1.18, 3.43)	19.3 %
UBA-behaviour-RR	20/17		
Target population engaged in intervention (yes vs. no)		1.47 (1.02, 2.11)	27.4 %

*RCT* randomized controlled trials, *UBA* uncontrolled before-and-after studies, *SMD* standardized mean difference (Hedges’ *g*), *RR* relative risk, *CI* confidence interval

^a^Adjusted *R* ^2^ refers to the proportion of between-study variance accounted for by the model

### Sensitivity analyses

The sensitivity analysis of imputing different correlation values for combining multiple outcomes in a study revealed that the analyses were robust to these values and changing the correlations had a negligible impact on the results (Additional file 11). However, for RCTs and NRTs of continuous behaviour outcomes, and for all uncontrolled before-and-after study continuous outcomes, sensitivity analyses revealed that selection of the imputed pre-post correlation in some cases changed the significance of estimates or changed estimates by >20 % (Additional file 12). In these cases, uncertainty in the meta-analyses estimates due to imputation of the pre-post correlation value was accounted for by appropriately downgrading the estimates in the GRADE assessment (Table 1). No consistent trend or impact on average meta-analysis estimates was noted when comparing pre-to-post vs. pre-to-follow-up measurements in studies where both sets of data were available (Additional file 13).

## Discussion

This review used a structured and transparent approach to identify and synthesize available evidence on the effectiveness of food safety education for consumers. We identified 17 RCTs (Additional file 6), which provide the highest evidence for determining causality and intervention effectiveness because the randomization process helps to control for unmeasured confounders that could otherwise influence the intervention effect [26, 49, 50]. However, we also decided a priori to include non-randomized designs in this review, including uncontrolled before-and-after studies, to allow a more comprehensive and complete assessment of the available evidence in this area, recognizing that RCTs may not be feasible for many large-scale food safety education interventions [26, 50, 51]. For example, two RCTs of the effectiveness of the Expanded Food and Nutrition Education Program (EFNEP) to improve nutrition and food safety outcomes in low-income youth and adults used a ‘delayed intervention’ group instead of a traditional control group for this reason, reporting that key program staff and implementers were more likely to participate knowing that both groups would receive the intervention at the conclusion of the study [42, 52]. Even in this case, Townsend et al. (2006) noted that some control groups chose not to comply with their group assignment and still offered the intervention during their study [42], which highlights some of the practical challenges in implementing traditional RCTs in this area.

Eleven of the 17 RCTs in this review did not specify their method of randomization, and many RCTs and NRTs did not specify their method of sequence allocation or measures taken to blind participants, study personnel, and outcome assessors to the group allocation status, resulting in several unclear ratings for these risk-of-bias criteria (Table 2). The first criterion is important to ensure a proper randomization process is used that will balance unmeasured confounding variables across groups [26]. The blinding criteria noted above are important to prevent against differential treatment and assessment of outcomes in participants based on possible knowledge of their group assignment, particularly for subjective outcomes such as attitudes and self-reported behaviours [26]. However, we recognize that blinding is challenging and often not feasible to implement in the context of educational interventions [53], and we did not downgrade the overall risk-of-bias rating for study outcomes based solely on unclear ratings for these criteria. For some criteria high risk-of-bias ratings were noted for RCTs and NRTs mostly due to incomplete outcome data and selective reporting resulting from a large and imbalanced proportion of drop-outs in one of the intervention groups [54, 55], exclusion of some results from analysis [56, 57], omission of quantitative results for some non-significant findings [40, 54, 57], and in one case because the similarity of baseline characteristics between intervention groups could not be determined [58]. Future experimental research investigating the effectiveness of food safety education interventions should aim to conduct and report methods and findings in accordance with appropriate guidelines for RCTs (CONSORT) and NRTs (TREND) [59, 60]. An extension to the CONSORT guidelines is also planned for social and psychological interventions [53].

Two large, well-conducted RCTs (high GRADE rating) found that food safety education training and course interventions are effective at improving behaviour outcomes in children and youth (Table 3). Specifically, both Townsend et al. (2006) and Quick et al. (2013) reported that community-based EFNEP workshops and a web-based video game implemented in a classroom setting increased food safety behaviours in low-income youth and middle school children, respectively [42, 61]. Although comparatively less research was identified specifically targeting children and youth compared to adults, the evidence suggests that school and after-school programs could be an important intervention point to enhance the food safety behaviours of consumers at a young age. Two small RCTs (moderate GRADE rating) found that a dialogical (i.e. engaging) video message and an instructional written and graphical message about *Salmonella* improved food safety behavioural intentions in adults [62, 63], indicating that food safety messaging interventions may be effective for these outcomes. Behaviour outcomes provide a more direct measure of intervention effectiveness compared to knowledge and attitudes; however, most of the studies analyzed in this review measured self-reported behaviours, which can be subject to social desirability bias and can be overestimated compared to observed practices [64, 65]. Nevertheless, several researchers have reported consistent agreement between self-reported and observed behaviours, and between behavioural intentions and observed behaviours, in consumers [37, 66, 67]. The agreement between these measures likely depends at least partially on the validity and reliability of the measurement instrument used. Given that self-reported behaviour outcomes are more feasible to measure in practice, future primary research collecting these outcomes should use measurement tools that have been appropriately assessed for their psychometric properties and have good agreement with observed behaviours to ensure validity and reliability of the findings.

A moderate GRADE rating was determined for the meta-analysis of two NRT studies on the impact of educational training and course interventions on attitude outcomes in adults. Both studies were university-based, and investigated the impacts of social media training, distance education, and a traditional classroom lecture to improve food safety attitude scores in university students and staff [68, 69]. Changes in attitudes are important precursors to behaviour change, as they help to shape an individual’s views of the importance and need for change and impact their behavioural intentions [32, 33]. Although RCTs and NRTs captured in this review reported beneficial median intervention effects for other intervention-population-outcome combinations, the confidence in these results was less reliable and future studies are likely to change the magnitude and possibly the direction of the conclusions.

Fifty of the 79 total relevant studies in this review (63 %) used an uncontrolled before-and-after study design (i.e. pre-post testing in the same population with no separate control group). Although these studies on average found consistent positive effects for all intervention-population-outcome combinations, results were very heterogeneous. In addition, all outcomes reported in these studies received a very low GRADE rating, and many received an unclear overall risk-of-bias rating due to limited reporting of methodological details for one or more criteria. A major limitation of these studies is that the lack of a separate control group limits our ability to draw causal inferences about intervention effectiveness given the potential for secular changes and other external variables to influence the results between pre- and post-tests [49, 50]. Therefore, the results of these studies should not be used directly to inform decision-making on food safety education program development or implementation; instead, the primary utility of these studies lies in their ability to show ‘proof of concept’ for an intervention effect to inform more robust experimental designs [26, 49, 50]. As noted above, proof of concept was demonstrated for a wide variety of education interventions in multiple consumer populations, including educators, for all investigated outcomes, indicating that future research should build on these interventions ideally through well-conducted RCTs.

A significant intervention dose effect was identified in meta-regression for behaviour outcomes in RCTs. This result provides support that food safety training interventions with more than one session or lesson and media campaigns and messaging interventions that provide materials through more than one medium or exposure type (i.e. multifaceted interventions) can enhance consumer safe-food handling behaviour change. This finding corresponds with those of some individual studies captured within this review. For example, in an evaluation of a social media-based intervention in college students, Mayer et al. (2012) reported that exposure to the social media component (Facebook website) for at least 15 min/week, particularly when combined with a traditional course lecture, resulted in improved food safety knowledge, attitude, and behaviour outcomes [69]. In addition, several other studies reported that food safety outcomes improved in consumers with a greater number of training sessions administered [70, 71] or with exposure to multiple intervention messaging materials [72–74], although in some cases a threshold level was reached beyond which additional exposures (e.g. lessons) did not result in further improvements to the measured outcomes. Future RCTs on the effectiveness of food safety interventions for consumers should investigate further the potential impact of dose on intervention effectiveness.

Significant predictors of between-study heterogeneity were identified in two of the meta-regression models of outcomes in uncontrolled before-and-after studies. Studies published in a source other than a peer-reviewed journal (i.e. theses and reports) were more likely to report a beneficial intervention effect for dichotomous knowledge outcomes. This finding may indicate a publication bias, which usually indicates that authors are more likely to publish positive and significant results in peer-reviewed journal articles, but in this case could reflect that findings were not subsequently published in a peer-reviewed journal due to a lack of perceived importance of the results or ability or desire to publish [75]. This finding highlights the importance of including gray literature sources such as theses and reports in systematic reviews and meta-analysis to ensure a more complete assessment of the available evidence. The other significant meta-regression finding indicated that studies that engaged their target population in the development, implementation, and/or evaluation of the intervention were more likely to report a beneficial intervention effect for dichotomous behaviour outcomes. This result corresponds with a recent systematic review that found that interventions using community engagement approaches positively impacted health behaviours and outcomes in a variety of different public health contexts [76]. Moreover, previous research has shown that consumers prefer food safety education interventions that are interactive and engaging [77, 69].

Food safety behaviours are often subdivided into specific behavioural constructs such as personal hygiene, adequate cooking of foods, avoiding cross-contamination, keeping foods at safe temperatures, and avoiding food from unsafe sources [78]. However, our ability to investigate these concepts in detail was limited by the availability and reporting of primary research in the various data subsets, as many studies only reported overall scores or scales. In addition, for similar reasons, attitudes were not further subdivided into key constructs from relevant behaviour change theories such as the Theory of Planned Behaviour, The Stages of Change Theory (Transtheoretical Model), and the Heath Belief Model [32, 33, 79]. For example, constructs such as self-efficacy, perceived behavioural control, risk perceptions (e.g. perceived susceptibility/severity of illness), and subjective norms have all been associated more specifically with intended and reported food safety behaviours [80, 81, 67]. Future experimental research should investigate and report further on various theoretical constructs and their relationship with specific food safety behaviours.

Most of the meta-analysis data subgroups contained significant heterogeneity that was unexplainable by variables examined in meta-regression models. Due to the limited availability of studies within each subgroup, our power to identify potential predictors of between-trial heterogeneity in meta-regression was limited. There are several additional population, intervention, outcome, and study design characteristics that could have influenced this heterogeneity but we were not able to investigate in this analysis. For example, the wide variety of outcome measurement instruments and scales used across studies could have contributed to this variation. For this reason, we used the SMD outcome measure in meta-analyses of continuous data; although this measure does not allow us to determine whether heterogeneity between trials is a true reflection of different participant outcomes or due to differences in how the outcomes were measured [26, 38]. Another limitation of this review is that correlation values for most studies were not reported and we had to impute plausible values from other comparable studies to allow for meta-analysis. Sensitivity analyses indicated this was a potential concern for some outcomes of studies that used an imputed value of the pre-post correlation. Based on our findings, correlation values are often not reported in primary research articles in this research area, but with increasing opportunities to publish supplementary materials online, we encourage primary research authors to make these data available in future publications. Finally, it is possible that we could have missed some relevant studies if they were not captured by our search algorithm. However, we implemented a comprehensive verification strategy in an attempt to minimize this potential bias.

## Conclusions

The effectiveness of food safety education interventions to improve consumer knowledge, attitude, and behaviour outcomes was evaluated in multiple experimental study designs conducted in developed countries. We identified a moderate to high confidence in intervention effectiveness for some outcomes in RCTs and NRTs, including: community- and school-based educational training on behaviours of children and youth; video and written instructional messaging on behavioural intentions in adults; and university-based education on attitudes of students and staff. While most RCTs and NRTs indicated a positive intervention effect for other outcomes, risk-of-bias and reporting limitations and the presence of significant heterogeneity between studies resulted in low and very low confidence in these findings. Meta-regression results showed a positive dose-response effect on behaviour outcomes in RCTs and a positive impact of engaging the target population in the intervention on knowledge outcomes in uncontrolled before-and-after studies, warranting further investigation. Many different education interventions were found to be effective in uncontrolled before-and-after studies at improving consumer food safety outcomes in a variety of contexts; future research should build upon this knowledge with well-conducted and reported RCTs. Future research is also needed to investigate further the factors contributing to the heterogeneity in intervention effectiveness across studies.

## Additional files

Additional file 1:
**PRISMA checklist.** (DOCX 30 kb) Additional file 2:
**Full details of the search strategy.** (DOCX 33 kb) Additional file 3:
**A copy of all review forms.** (DOCX 63 kb) Additional file 4:
**Correlation values from previous studies.** (XLSX 11 kb) Additional file 5:
**List of studies with more than two intervention and control groups.** (DOCX 25 kb) Additional file 6:
**Citation list of all 77 relevant articles.** (XLS 44 kb) Additional file 7:
**Summary table of PICO characteristics for each relevant article.** (XLS 65 kb) Additional file 8:
**Detailed within-study risk-of-bias assessment results.** (XLS 92 kb) Additional file 9:
**Forest plots for each meta-analysis subgroup.** (DOCX 527 kb) Additional file 10:
**Detailed GRADE assessment results.** (XLS 31 kb) Additional file 11:
**Sensitivity analysis of imputing different correlations for combining multiple outcome measures within a study.** (XLS 29 kb) Additional file 12:
**Sensitivity analysis of imputing different pre-post correlations for paired meta-analyses.** (XLS 27 kb) Additional file 13:
**Sensitivity analysis of comparing meta-analysis estimates for pre-post vs. pre-follow-up measurements.** (XLS 26 kb)
