# Supplementary material for: A systematic review and meta-analysis of the effectiveness of food safety education interventions for consumers in developed countries
Source: BMC Public Health. 2015 Aug 26;15:822. doi: 10.1186/s12889-015-2171-x (PMC4548310; doi:10.1186/s12889-015-2171-x)
Supplement: Additional file 2: — Full details of the search strategy. (DOCX 33 kb) [file 12889_2015_2171_MOESM2_ESM.docx]

Additional File 2: Full Search Strategy

The search algorithm as implemented in each bibliographic database was developed by reviewing and combing key terms identified in the titles and abstracts of 15 pre-selected relevant articles (list available upon request from corresponding author). The search algorithm was then pretested in Scopus and revised as appropriate to ensure that all pre-selected articles were captured. The full algorithm as implemented in each bibliographic database is shown in the tables below.

*Detailed search algorithms for each bibliographic database*

| **Date** | May 20, 2014 |
| --- | --- |
| **Performed by** | Ian Young |
| **Platform/Interface** | OvidSP |
| **Databases** | CAB Abstracts (1973-2014 week 19)  Agricola (1970-May 2014)  Food Science and Technology Abstracts (1969-2014 May Week 2)  PsycINFO (1806-2014 May Week 2) |
| **Institution** | Public Health Agency of Canada |
| **Search string:** | *(("food safety" OR "food-borne" OR foodborne OR foodbourne OR “food-bourne” OR "food handling" OR "food preparation" OR "food poisoning" OR “food hygiene” OR “safe food”) AND (consumer* OR client* OR population OR public OR people OR person OR persons OR individual OR individuals OR student OR students OR children OR youth* OR adolescent* OR teen* OR parent OR parents OR mother* OR father* OR adult OR adults OR women OR female* OR communit* OR families OR family OR household* OR domestic OR volunteer OR volunteers OR home OR homes OR school* OR campus OR university OR universities OR college*) AND (campaign* OR strateg* OR program* OR messag* OR intervention* OR technology OR teach* OR curriculum OR workshop* OR initiative* OR educat* OR communicat* OR information OR media OR brochure OR pamphlet OR learn* OR instruction* OR train* OR label* OR internet) AND (awareness OR knowledge OR practice* OR behaviour* OR behavior* OR attitude* OR perception* OR preference* OR learn* OR belief* OR acceptance))*  in **Article title**  **OR**  in **Abstract**  **OR**  in **Descriptors** |
| **Hits** | 5219 |
| **Limits** | None |

| **Date** | May 20, 2014 |
| --- | --- |
| **Performed by** | Ian Young |
| **Platform/Interface** | ProQuest |
| **Databases** | ProQuest Public Health (1972-2014) |
| **Institution** | Public Health Agency of Canada |
| **Search string:** | *(("food safety" OR "food-borne" OR foodborne OR foodbourne OR “food-bourne” OR "food handling" OR "food preparation" OR "food poisoning" OR “food hygiene” OR “safe food”) AND (consumer* OR client* OR population OR public OR people OR person OR persons OR individual OR individuals OR student OR students OR children OR youth* OR adolescent* OR teen* OR parent OR parents OR mother* OR father* OR adult OR adults OR women OR female* OR communit* OR families OR family OR household* OR domestic OR volunteer OR volunteers OR home OR homes OR school* OR campus OR university OR universities OR college*) AND (campaign* OR strateg* OR program* OR messag* OR intervention* OR technology OR teach* OR curriculum OR workshop* OR initiative* OR educat* OR communicat* OR information OR media OR brochure OR pamphlet OR learn* OR instruction* OR train* OR label* OR internet) AND (awareness OR knowledge OR practice* OR behaviour* OR behavior* OR attitude* OR perception* OR preference* OR learn* OR belief* OR acceptance))*  in **Document title**  **OR**  in **Abstract**  **OR**  in **Subject heading (all)** |
| **Hits** | 914 |
| **Limits** | None |

| **Date** | May 20, 2014 |
| --- | --- |
| **Performed by** | Ian Young |
| **Platform/Interface** | ProQuest |
| **Databases** | ERIC (1966-2014) |
| **Institution** | Public Health Agency of Canada |
| **Search string:** | *(("food safety" OR "food-borne" OR foodborne OR foodbourne OR “food-bourne” OR "food handling" OR "food preparation" OR "food poisoning" OR “food hygiene” OR “safe food”) AND (consumer* OR client* OR population OR public OR people OR person OR persons OR individual OR individuals OR student OR students OR children OR youth* OR adolescent* OR teen* OR parent OR parents OR mother* OR father* OR adult OR adults OR women OR female* OR communit* OR families OR family OR household* OR domestic OR volunteer OR volunteers OR home OR homes OR school* OR campus OR university OR universities OR college*) AND (campaign* OR strateg* OR program* OR messag* OR intervention* OR technology OR teach* OR curriculum OR workshop* OR initiative* OR educat* OR communicat* OR information OR media OR brochure OR pamphlet OR learn* OR instruction* OR train* OR label* OR internet) AND (awareness OR knowledge OR practice* OR behaviour* OR behavior* OR attitude* OR perception* OR preference* OR learn* OR belief* OR acceptance))*  in **Document title**  **OR**  in **Abstract**  **OR**  in **Subject heading** |
| **Hits** | 349 |
| **Limits** | None |

| **Date** | May 20, 2014 |
| --- | --- |
| **Performed by** | Bhairavi Sivaram |
| **Platform/Interface** | ProQuest |
| **Databases** | ProQuest Dissertations & Theses A&I‎ (1743-2014) |
| **Institution** | University of Guelph |
| **Search string:** | *(("food safety" OR "food-borne" OR foodborne OR foodbourne OR “food-bourne” OR "food handling" OR "food preparation" OR "food poisoning" OR “food hygiene” OR “safe food”) AND (consumer* OR client* OR population OR public OR people OR person OR persons OR individual OR individuals OR student OR students OR children OR youth* OR adolescent* OR teen* OR parent OR parents OR mother* OR father* OR adult OR adults OR women OR female* OR communit* OR families OR family OR household* OR domestic OR volunteer OR volunteers OR home OR homes OR school* OR campus OR university OR universities OR college*) AND (campaign* OR strateg* OR program* OR messag* OR intervention* OR technology OR teach* OR curriculum OR workshop* OR initiative* OR educat* OR communicat* OR information OR media OR brochure OR pamphlet OR learn* OR instruction* OR train* OR label* OR internet) AND (awareness OR knowledge OR practice* OR behaviour* OR behavior* OR attitude* OR perception* OR preference* OR learn* OR belief* OR acceptance))*  in **Title**  **OR**  in **Abstract**  OR  in **Subject heading (all)** |
| **Hits** | 408 |
| **Limits** | None |

| **Date** | May 20, 2014 |
| --- | --- |
| **Performed by** | Bhairavi Sivaram |
| **Platform/Interface** | Scopus |
| **Databases** | Scopus (1823-2014) |
| **Institution** | Public Health Agency of Canada |
| **Search string:** | *(("food safety" OR "food-borne" OR foodborne OR foodbourne OR “food-bourne” OR "food handling" OR "food preparation" OR "food poisoning" OR “food hygiene” OR “safe food”) AND (consumer* OR client* OR population OR public OR people OR person OR persons OR individual OR individuals OR student OR students OR children OR youth* OR adolescent* OR teen* OR parent OR parents OR mother* OR father* OR adult OR adults OR women OR female* OR communit* OR families OR family OR household* OR domestic OR volunteer OR volunteers OR home OR homes OR school* OR campus OR university OR universities OR college*) AND (campaign* OR strateg* OR program* OR messag* OR intervention* OR technology OR teach* OR curriculum OR workshop* OR initiative* OR educat* OR communicat* OR information OR media OR brochure OR pamphlet OR learn* OR instruction* OR train* OR label* OR internet) AND (awareness OR knowledge OR practice* OR behaviour* OR behavior* OR attitude* OR perception* OR preference* OR learn* OR belief* OR acceptance))*  in **Title**  **OR**  in **Abstract**  **OR**  **Key words** |
| **Hits** | 5309 |
| **Limits** | None |

| **Date** | May 20, 2014 |
| --- | --- |
| **Performed by** | Bhairavi Sivaram |
| **Platform/Interface** | PubMed |
| **Databases** | PubMed (1950-2014) |
| **Institution** | Public Health Agency of Canada |
| **Search string:** | *(("food safety" OR "food-borne" OR foodborne OR foodbourne OR “food-bourne” OR "food handling" OR "food preparation" OR "food poisoning" OR “food hygiene” OR “safe food”) AND (consumer* OR client* OR population OR public OR people OR person OR persons OR individual OR individuals OR student OR students OR children OR youth* OR adolescent* OR teen* OR parent OR parents OR mother* OR father* OR adult OR adults OR women OR female* OR communit* OR families OR family OR household* OR domestic OR volunteer OR volunteers OR home OR homes OR school* OR campus OR university OR universities OR college*) AND (campaign* OR strateg* OR program* OR messag* OR intervention* OR technology OR teach* OR curriculum OR workshop* OR initiative* OR educat* OR communicat* OR information OR media OR brochure OR pamphlet OR learn* OR instruction* OR train* OR label* OR internet) AND (awareness OR knowledge OR practice* OR behaviour* OR behavior* OR attitude* OR perception* OR preference* OR learn* OR belief* OR acceptance))*  in **Title**  **OR**  in **Abstract**  **OR**  in **MeSH Terms** |
| **Hits** | 3090 |
| **Limits** | None |

| **Date** | May 20, 2014 |
| --- | --- |
| **Performed by** | Bhairavi Sivaram |
| **Platform/Interface** | EBSCOHost |
| **Databases** | CINAHL Plus with Full Text (1937-2014) |
| **Institution** | University of Guelph |
| **Search string:** | *(("food safety" OR "food-borne" OR foodborne OR foodbourne OR “food-bourne” OR "food handling" OR "food preparation" OR "food poisoning" OR “food hygiene” OR “safe food”) AND (consumer* OR client* OR population OR public OR people OR person OR persons OR individual OR individuals OR student OR students OR children OR youth* OR adolescent* OR teen* OR parent OR parents OR mother* OR father* OR adult OR adults OR women OR female* OR communit* OR families OR family OR household* OR domestic OR volunteer OR volunteers OR home OR homes OR school* OR campus OR university OR universities OR college*) AND (campaign* OR strateg* OR program* OR messag* OR intervention* OR technology OR teach* OR curriculum OR workshop* OR initiative* OR educat* OR communicat* OR information OR media OR brochure OR pamphlet OR learn* OR instruction* OR train* OR label* OR internet) AND (awareness OR knowledge OR practice* OR behaviour* OR behavior* OR attitude* OR perception* OR preference* OR learn* OR belief* OR acceptance))*  in **Title**  **OR**  in **Abstract**  OR  in **Subject** |
| **Hits** | 414 |
| **Limits** | None |

*Citation List of 30 Articles used in Search Verification*

Abbot JM, Policastro P, Bruhn C, Schaffner DW, Byrd-Bredbenner C. 2012. Development and evaluation of a university campus-based food safety media campaign for young adults. J Food Prot 75(6):1117-24.

Al-Sakkaf A. 2013. Domestic food preparation practices: a review of the reasons for poor home hygiene practices. Health Promot Int. doi: 10.1093/heapro/dat051.

Arnold CG and Sobal J. 2000. Food practices and nutrition knowledge after graduation from the expanded food and nutrition education program (EFNEP). J Nutr Educ 32(3):130-138.

Bearth A, Cousin M, Siegrist M. 2013. Uninvited guests at the table – a consumer intervention for safe poultry preparation. J Food Saf 33(4):394-404.

Bruhn CM. 2009. History of consumer food safety education focus on beef: impact on risk of foodborne illness. Food Protection Trends 29(11):793-799.

Byrd-Bredbenner C, Maurer J, Wheatley V, Schaffner D, Bruhn C, Blalock L. 2007. Food safety self-reported behaviors and cognitions of young adults: results of a national study. Journal of Food Protection 70(8):1917-1926.

Campbell ME, Gardner CE, Dwyer JJ, Isaacs SM, Krueger PD, Ying JY. 1998. Effectiveness of public health interventions in food safety: a systematic review. Can J Public Health 89(3):197-202.

Cates SC, Carter-Young HL, Conley S, O'Brien B. 2004. Pregnant women and listeriosis: preferred educational messages and delivery mechanisms. J Nutr Educ Behav 36(3):121-127.

Chen G, Kendall PA, Hillers VN, Medeiros LC. 2010. Qualitative studies of the food safety knowledge and perceptions of transplant patients. J Food Prot 73(2):327-35.

Dharod JM, Perez-Escamilla R, Bermudez-Millan A, Segura-Perez S, Damio G. 2004. Influence of the fight BAC! food safety campaign on an urban Latino population in Connecticut. J Nutr Educ Behav 36(3):128-132.

Dollahite JS, Pijai EI, Scott-Pierce M, Parker C, Trochim W. 2014. A randomized controlled trial of a community-based nutrition education program for low-income parents. J Nutr Educ Behav 46(2):102-9.

Faccio E, Costa N, Losasso C, Cappa V, Mantovani C, Cibin V, Andrighetto I, Ricci A. 2013. What programs work to promote health for children? exploring beliefs on microorganisms and on food safety control behavior in primary schools. Food Control 33(2):320-329.

Henley SC. 2013. "Don't wash your chicken!" results of an interdisciplinary approach to reduce incidence of infectious foodborne diseases. *PhD thesis.* Drexel University.

Jacob C, Mathiasen L, Powell D. 2010. Designing effective messages for microbial food safety hazards. Food Control 21(1):1-6.

Mann V, DeWolfe J, Mowatt JF, Hart R, Hollands H, LaFrance R, Lee M, Ying J. 2001. Systematic review of the effectiveness of food safety interventions. Ontario, Canada: Public Health Research, Education and Development Program. [www.ephpp.ca/PDF/2001_**Food**%20**Safety**_Summ.pdf](http://www.ephpp.ca/PDF/2001_Food%20Safety_Summ.pdf)

Medeiros L, Hillers V, Kendall P, Mason A. 2001. Evaluation of food safety education for consumers. J Nutr Educ 33 Suppl 1:S27-34.

Milton A and Mullan B. 2010. Consumer food safety education for the domestic environment: a systematic review. Br Food J 112(9):1003-22.

Mullan B. 2011. Using social-cognition models to predict and design interventions to modify consumers’ safe food handling behaviour. Walsch, MB (Ed). In: Food Supplies and Food Safety. Nova Science Publishers Inc. pp. 151-187.

Nesbitt A, Thomas MK, Marshall B, Snedeker K, Meleta K, Watson B, Bienefeld M. 2014. Baseline for consumer food safety knowledge and behaviour in Canada. Food Control 38:157-173.

Powell DA, Surgeoner BV, Wilson SM, Chapman BJ. 2007. The media and the message: risk analysis and compelling food safety information from farm-to-fork. Australian Journal of Dairy Technology 62(2):55-59.

Quick V, Corda KW, Chamberlin B, Schaffner DW, Byrd-Bredbenner C. 2013. Ninja kitchen to the rescue: evaluation of a food safety education game for middle school youth. Br Food J 115(5):686-99.

Redmond EC and Griffith CJ. 2003. Consumer food handling in the home: a review of food safety studies. J Food Prot 66(1):130-161.

Redmond E, Griffith C, Redmond EC, Griffith CJ. 2006. A pilot study to evaluate the effectiveness of a social marketing-based consumer food safety initiative using observation. Br Food J 108(9):753-770.

Redmond EC and Griffith CJ. 2004. Consumer perceptions of food safety risk, control and responsibility. Appetite 43(3):309-13.

Snow MM. 2012. The effects of educational messaging within the expanded food and nutrition education program. *PhD thesis.* The University of Utah.

Takeuchi MT, Edlefsen M, McCurdy SM, Hillers VN. 2005. Educational intervention enhances consumers' readiness to adopt food thermometer use when cooking small cuts of meat: an application of the transtheoretical model. J Food Prot 68(9):1874-83.

Wang D and Stewart D. 2013. The implementation and effectiveness of school-based nutrition promotion programmes using a health-promoting schools approach: a systematic review. Public Health Nutr 16(6):1082-100.

Wilcock A, Pun M, Khanona J, Aung M. 2004. Consumer attitudes, knowledge and behaviour: a review of food safety issues. Trends Food Sci Technol 15(2):56-66.

Wildemann TM. 2006. Communicating risks of foodborne diseases. Central European Journal of Medicine 1(1):69-80.

Yarrow L, Remig VM, Higgins MM. 2009. Food safety educational intervention positively influences college students' food safety attitudes, beliefs, knowledge, and self-reported practices. J Environ Health 71(6):30-5.

*List of 24 Organization Websites Searched for Grey Literature*

1. United States Department of Agriculture (USDA)-Food Safety and Inspection Service
2. US Food and Drug Administration (FDA)
3. Council of State and Territorial Epidemiologists
4. US Centres for Disease Control (CDC)
5. European Food Safety Authority
6. European Centre for Disease Prevention and Control
7. International Union for Health Promotion and Education
8. Coalition of Health Communication
9. Food and Agriculture Organization of the United Nations (FAO)
10. World Health Organization (WHO)
11. National Institute for Public Health and the Environment, Netherlands
12. Institute of Environmental Science and Research (ESR)
13. Public Health Agency of Canada (PHAC)
14. Health Canada
15. National Collaborating Centre for Environmental Health
16. BC Centre for Disease Control
17. Public Health Ontario (PHO)
18. Public Health and Epidemiology Report Ontario (PHERO)
19. Public Health Research, Education & Development (PHRED)
20. Canadian Partnership for Consumer Food Safety Education
21. Public Health Grey Literature Database
22. Public Opinion Research and Evaluation Database (Canada)
23. Public Opinion Research Reports (Canada)
24. Research Triangle Institute (RTI) International
