# Supplementary material for: A systematic review and meta-analysis of the effectiveness of food safety education interventions for consumers in developed countries
Source: BMC Public Health. 2015 Aug 26;15:822. doi: 10.1186/s12889-015-2171-x (PMC4548310; doi:10.1186/s12889-015-2171-x)
Supplement: Additional file 3: — A copy of all review forms. (DOCX 63 kb) [file 12889_2015_2171_MOESM3_ESM.docx]

Additional File 3: Review Forms

Relevance Screening Form

| **Question** | **Options** | **Definitions/additional notes** |
| --- | --- | --- |
| 1. Does the citation describe primary research investigating the effectiveness of food safety education programs or interventions for consumers? | - Yes - No | Food safety:  Practicing safe handling, preparing, storing, and consumption of food in order to prevent foodborne illness (e.g. personal hygiene and hand-washing, avoiding cross contamination, adequate cooking of food, time-temperature control, avoiding food from unsafe sources). Includes microbial, chemical and/or physical hazards that could cause foodborne illness or other adverse health effect.  Food safety education programs or interventions:  For the purposes of this study, refers to ***consumer-level*** educational food safety materials (e.g. pamphlets, brochures, videos), food safety training/classes (e.g. workshops, online modules/course), food safety communications (e.g. media campaigns, public messaging, social marketing), and food safety curriculum/lessons in a school or academic setting.  Consumers:  The population of interest is consumers, including the general public, home cooks, household food preparers, high risk populations (e.g. immune compromised, pregnant women, elderly), students, ethnic groups, and volunteer cooks for special food events or bake sales hosted by religious organizations, service clubs or fraternal organizations. Include any studies on *educators of consumers* (e.g. train the trainer).  Include   - Research on any qualitative or quantitative outcome measure of intervention effectiveness (e.g. self-reported or observed changes in practices, attitude or knowledge; frequency of foodborne illness; bacterial counts) - All study designs, publication dates, and publication types, including grey literature (e.g. public opinion research) - Qualitative studies that investigate consumers' preferences/attitudes towards receiving educational messaging/materials, etc., even if no specific intervention is identified - Studies evaluating questionnaire instruments for use in food safety education interventions of consumers as long as there is primary data collection - Studies published in English, French or Spanish - Studies where you “can’t tell” the relevance and suspect it could be relevant   Exclude   - Quantitative studies with *no intervention* mentioned (e.g. a survey of the prevalence of consumer food safety practices with no associated intervention) - Studies *not directly related to food safety* (e.g. generic hand-washing only). Studies on water quality/safety should also be excluded unless reported in a food safety context (e.g. safe water for food preparation). - Research on GMOs and food allergies. - Food safety studies *not conducted at the consumer level* (e.g. primary food processing/manufacturing) - Studies focusing on *interventions directed at food handlers* (e.g. food handler training) - *Non-educational interventions* (e.g. food premise inspections, public disclosure of inspection scores) |
| 2. Check this box if you selected no above and the abstract describes a review or commentary article about food safety education for consumers | - Potentially relevant review / commentary article | DO NOT address conflicts for this question – it is included for our informational purposes only to help with search verification. |

Article Characterization and Charting Form

| **Question** | **Options** | **Comments** |
| --- | --- | --- |
| 1) Is this primary research investigating the effectiveness of food safety education programs or interventions for consumers published in English, French, or Spanish? | Yes, primary quantitative research  Yes, primary qualitative research  Yes, primary mixed-method research  No, specify reason(s) for exclusion:   - Not relevant : _________ - Other language: _________ - Other:____________   *If “no” is selected, submit form without proceeding further.* | **Food safety education programs or interventions:** For the purposes of this study, refers to ***consumer-level*** educational food safety materials (e.g. pamphlets, brochures, videos), food safety training (e.g. workshops, online modules/course), food safety communications (e.g. media campaigns, public messaging, social marketing), and food safety curriculum/lessons in a school or academic setting.  **Consumers:** The population of interest is consumers, including the general public, home cooks, high risk populations (e.g. immune compromised, pregnant women, elderly), students, ethnic groups, and volunteer cooks for special food events or bake sales hosted by religious organizations, service clubs or fraternal organizations. Include any studies on *educators of consumers* (e.g. train the trainer). Research on ***food handlers*** should be **excluded**.  **Primary research**: a study where the authors collected and analyzed their own data – may use quantitative or qualitative methods or both to investigate the research question and report original results.  **Quantitative primary research:** Investigator(s) collected samples or data themselves for analysis- usually a numerical summarization (e.g. cross-sectional studies, surveys, case-control studies)  **Qualitative research**: Aimed at understanding social phenomena, exploring issues, and answering questions of “why” and “how” as opposed to numerical summarization of results. Not usually generalizable to a whole population. Examples: focus groups, interviews.  **Mixed-method research:** Conduct of qualitative and quantitative methods in the same study. A larger sample usually means the results are more generalizable. (e.g. focus groups and a quantitative survey/questionnaire)  **Exclude:**   - Studies with *no intervention* mentioned (e.g. a survey of the prevalence of consumer food safety practices with no intervention) - Studies with *no relevant outcomes* (e.g. willingness to pay, food purchase decisions, etc.) - Studies *not directly related to food safety* (e.g. generic hand-washing only) - Food safety studies *not conducted at the consumer level* (e.g. primary food processing/manufacturing) - Studies focusing on *interventions directed at food handlers* (e.g. food handler training) - *Non-educational interventions* (e.g. food premise inspections, public disclosure of inspection scores) - Studies in languages other than English, French, or Spanish |
| 2) What is the publication year of this article? | _______ | Enter year |
| 3) What type of document is this article? | Journal article  Conference proceedings  Government or research report  Thesis  Book or book chapter  Other, please specify:_____ |  |
| 4) What is the article language? | English  French  Spanish |  |
| 5) Where was the study conducted?  *(Check all that apply)* | North America:   - Canada, province: ____ - USA, state: ______ - Mexico, state: _______   Europe:______________  Australasia:_____  Central and South America/ Caribbean:________  Asia:_____________________  Africa:____________________  Not stated | **North America:** Canada, USA and Mexico [please specify province or state only and use full name (*e.g.* USA, New York; or if two states: USA, New York, Florida) DO NOT INCLUDE REGION etc.]  **South America/ Caribbean:** Caribbean, and all of south America. [Please specify country only and use full name *e.g.* Columbia; do not include region etc.]  **Europe:** includes, Belarus, Latvia, Ukraine, Estonia, Cyprus & west (inc. Iceland and Greenland) [please specify country only and use full name (*e.g.* England) do not include region etc.] If study done in the United Kingdom, please state the country rather than UK.  **Asia:** Russia, Turkey, middle eastern countries and east [please specify country only and use full name *e.g.* Japan do not include region etc.]  **Australasia** is limited to Australia, New Guinea, New Zealand, New Caledonia, and neighbouring islands, including the Indonesian islands from Lombok and Sulawesi eastward. [Please specify country only and use full name *e.g.* New Zealand; do not include region etc.] |
| 6) What is the study design?  *(Check all that apply)* | Observational study:   - Cross-sectional - Cohort - Case-control - Prevalence survey - Other:______________   Experimental study:   - Randomized controlled trial (RCT) - Non-randomized controlled trial - Controlled before-and-after study (CBA) - Uncontrolled before-and-after study - Interrupted time series (ITS) - Other:_______________   Qualitative study, specify:___  Process evaluation  Other, please specify:_______ | **If more than one design, report ONLY study design(s) relevant to the research question.**  **Observational study**: Assignment of subjects into a treated group versus a control group is outside the control of the investigator.   - **Cross-sectional:** Examines the relationship of a risk factor and outcome (disease) at a point in time on representative samples of the target population. - **Cohort study**: is a study in which individuals with differing exposures to a suspected risk factor are observed through time for occurrence of an outcome - **Case-control study**: compares exposure to the risk factor in subjects who have an outcome (the 'cases') with subjects who do not have the outcome, but are otherwise similar (the 'controls') and drawn from the same sampling frame. - **Prevalence survey:** Measurement of an outcome at a point in time but doesn’t measure or investigate potential predictors   **Experimental study:** Each subject is assigned to a treated group or a control group before the start of the treatment   - **Controlled trial:** an experimental study in which people are allocated to intervention/comparison groups and evaluated for outcomes. Randomized (RCT) if authors specifically indicate random allocation of treatment/control. - **Controlled before-and-after (CBA) study:** A study in which observations are made before and after the implementation of an intervention, both in a group that receives the intervention and in a control group that does not. - **Uncontrolled before-and-after study:** observations are made on a population before and after receiving an intervention. - **Interrupted-time-series (ITS) study:** A study that uses observations at *multiple time points* before and after an intervention (the ‘interruption’). Differs from uncontrolled before-after study in that multiple measurements (≥3) are made before AND after the intervention.   **Qualitative study:** Aimed at understanding social phenomena, exploring issues, and answering questions of “why” and “how.” Example designs include descriptive, grounded theory, phenomenology, ethnography. ***Please specify the design/methodology that is identified by the author, and if none is identified explicitly then indicate “not specified”.***  **Process evaluation:** systematic method of collecting and analyzing data to determine if programs/policies are being implemented as intended. |
| 7) Did the study include a control group? | Yes, independent control group  Yes, historical control group  Yes, internal control group  Yes, cross-over design  No  N/a – qualitative study | **Cross-over:** Participants cross from intervention to control group and vice versa at least once during the study.  **Internal control:** Measurements on the same group of individuals before and after receiving the intervention.  **Historical control**: Using data collected from individuals in past studies or available old records/databases |
| 8) When was the study conducted? | ___________  Not reported | Please specify year/month to year/month if available (do not extract days) [follow format example: 2000/06-2000/08] |
| 9) What was the name of the intervention? | ___________  Not reported  N/a – qualitative study |  |
| 10) What types of interventions were investigated? | Training workshop/session  Preschool to high school course  University/college course  Media campaign/social marketing/  other messaging  Other, specify:________  N/a – qualitative study | **Media campaign/social marketing:** Messaging that is intended to target a large group of audience through the traditional broadcast media, print, and internet media e.g. radio, TV, websites, social media; **other messaging:** includes programs or targeted messages offered by public health agencies and other sources (e.g. special messaging for pregnant women or cancer patients in doctor’s offices). Also includes food product labels and recipe books/cards. |
| 11) How was the intervention delivered? | In-person:   - One-to-one - Group   Online/web-based modules  Print media  TV  Radio  Multimedia  Website information  Social media  Product labels  Recipe books/cards  Other:_________________  Not reported  N/a – qualitative study | **Print media:** e.g. newspapers, newsletters, brochures, posters.  **Multimedia:** Any combination of print/text, audio, images, video, and/or interactive activities.  **Social media:** Interactive exchange of information in virtual communities and networks (e.g. forums, Facebook, Twitter, YouTube). |
| 12) What were the contents/topics covered by the intervention? | General/background food safety  Personal hygiene  Adequate cooking of foods  Avoiding cross-contamination  Time-temperature control  Avoiding food from unsafe sources/high-risk foods  Washing fruits/vegetables  Food spoilage  Other:______________  Non-food safety related topics:   - Nutrition - Food security - Other:__________   Not specified  N/a – qualitative study | **General/background food safety:** Includes topics such as foodborne pathogens/other hazards, illness/outbreaks, epidemiology, symptoms, diagnosis, treatment, etc.  **Personal hygiene:** hand-washing, avoiding food preparation when ill.  **Cooking:** cooking temperatures, use of thermometers when cooking.  **Cross-contamination:** cleaning of food preparation surfaces and equipment, separation of raw from cooked/RTE foods, safe water for food preparation, and protection of food from pests.  **Time-temperature control:** maintaining refrigerator temperatures, reheating and defrosting.  **Food from unsafe sources/high risk foods:** e.g. raw milk consumption, uncooked seafood, ungraded/raw eggs, unpasteurized juices.  **Food spoilage:** best before/use dates, expiry dates, food disposal, food storage.  * Based on constructs identified by Medeiros et al., 2001, JNE, 33(2), 108-111.  **Food security:** food access and availability |
| 13) Did the intervention focus on specific food products and/or hazards? | Yes, specify:   - Hazards:_________ - Products:__________   No / not specified  N/a – qualitative study | **Only answer YES if there is an explicit/clear focus on one or more KEY products/hazards.**  E.g. interventions could focus solely on practices to prevent illness from *Listeria* through consumption of ready-to-eat meats. |
| 14) Was the intervention design informed or based on a theory or model of human behaviour? | Yes, specify:   - Health Belief Model - Stages of Change Theory/Transtheoretical Model - Theory of Planned Behaviour - Theory of Reasoned Action - Health Action Process Approach - Social Marketing - Diffusion of Innovations - PRECEDE-PROCEED model - Social Learning/Cognitive Theory - Other:_______________   No / not specified  N/a – qualitative study | **Select any of the options ONLY IF AUTHORS EXPLICITLY INDICATED the theory was used.** |
| 15) If yes, were the results reported in the context of the theory or model of human behaviour? | Yes  No  N/a – qualitative study | **Select ‘Yes’ ONLY IF AUTHORS EXPLICITLY REPORTED the results in the context of the theory or model.**  **Examples:**  If informed by Stages of Change Theory, did results report how the intervention changed the participants’ reported/observed “stages of change” classification?  If Theory of Planned Behaviour, did the study identify a preconceived attitude, subjective norm, and/or perceived behaviour control among participants that was addressed by the intervention, which in turn altered their behaviour?  If Health Belief Model, did the results report that the intervention changed the participants' self-efficacy and/or perceived barriers/threats to changing their behaviour according to the Health Belief Model? |
| 16) Was the intervention informed by any formative primary research? | Yes, specify:   - Focus groups - Interviews - Survey - Other:_______________   No / not specified  N/a – qualitative study |  |
| 17) Did the intervention involve any facilitators or instructors? | Yes, specify:   - School teachers - Professors/lecturers - Extension professionals/ paraprofessional educators - Dieticians/nutritionists - Nurses - Public health inspectors/ environmental health officers - Members of the target population - Other:_______________   No / not specified  N/a – qualitative study | **Extension professional/Paraprofessionals educators**: personnel who conduct food safety training for the community in both informal and formal settings. They are not formal (school) teachers but conduct training in the field. |
| 18) Were members of the target population engaged in the intervention development, delivery and/or implementation? | Yes  No / not specified  N/a – qualitative study |  |
| 19) What was the intervention setting *(where was the intervention applied/delivered)*?*  * For qualitative studies – where was the data collection conducted? | Homes  School/university/college  Community/religious centres  Grocery stores/markets  Healthcare facility/medical clinic  Public places  Internet/web  Media  Other:________________  Not reported | **Public places:** transit, roads, squares, parks, government buildings open to the public. |
| 20) What was the intervention dose and duration? | Dose:_____________  Duration:________________  Not reported  N/a – qualitative study | **Dose:** refers to how often (and/or how much of) the intervention was applied (e.g. number of sessions conducted).  **Duration:** refers to how long the intervention was applied for.  NOTE: Where possible provide units of measurement (e.g. total hours, weeks, etc.) |
| 21) What was the target population of the study? | Consumers  Educators of consumers | **Consumers:** Includes the general public, home cooks, high risk populations (e.g. immune compromised, pregnant women, elderly), students, ethnic groups, and volunteer cooks for special food events or bake sales hosted by religious organizations, service clubs or fraternal organizations.  **Educators:** “train the trainer” studies –population’s educating/providing interventions to consumers are the focus. |
| 22) What were the key socio-demographic characteristics of the intervention target population *(or qualitative study participants)*? | Gender   - Male - Female   Age, specify mean/median and range:_____________   - Elderly - Adults - Youth - Children   Race/ethnicity   - Caucasian - Black - Indigenous - Hispanic/Latin American - Asian - Other:_____________   Geographic location:   - Urban - Rural   Socio-economic status (SES)   - Low income/SES status - High income/SES status - Other:______________   Students   - Pre-school/elementary students - High school students - College/university students   Occupation, specify:______  Education level, specify:______  Literacy level, specify:______  Immigration status, specify:___  Immuno-compromised  Pregnant  Parents/caregivers of children  Caregivers of elderly  Other:_________________  None reported | **Select options ONLY for key characteristics of the intervention target population AS SPECIFIED BY THE AUTHORS (in the intro/methods).**  **Age:** Specify the mean/median age and age range for both intervention and control groups (if available), otherwise for the whole sample population.  For the purposes of this review:  ***Elderly =*** Seniors, >65 years old  ***Adults =*** 20-65 years old  ***Youth =*** 15-19 years old  ***Children =*** 14 years and under  **Asian**: Includes Chinese, Japanese, Korean, South Asian (e.g. East Indian, Pakistani, Sri Lankan), and Southeast Asian (e.g. Vietnamese, Cambodian, Malaysian, Filipino).  **Immuno-compromised:** Examples of persons with weakened immune systems include those with AIDS; cancer and transplant patients who are taking certain immunosuppressive drugs; and those with inherited diseases that affect the immune system (e.g., congenital agammaglobulinemia, congenital IgA deficiency).  **Other:** specify any other social or cultural characteristics of the participants noted by the authors. |
| 23) What food safety outcomes were measured? | Attitudes/perceptions/beliefs  Knowledge/awareness  Behavioural intentions/motivations  Self-reported behaviours  Observed behaviours  Microbial prevalence/counts  Incidence of foodborne illness  Program participation/coverage rates  Economic measures  Other:_____________  N/a – qualitative study | **ONLY indicate food safety outcomes**, NOT other measures (e.g. nutrition outcomes). |
| 24) How often were outcome measurements assessed during the study? | Same frequency of measurement for all outcomes:   - Once - Twice - 3 times - >3 times, specify:__________   Not the same for all outcomes: ____  Not reported  N/a – qualitative study | Include any pre-intervention/baseline measurements in this count  **Not the same for all the articles**: Please specify outcome name as stated in Question 26 and the frequency of outcome measurement; if frequency of measurement is not reported for an outcome specify ‘NR’ for not reported [follow format example: Attitudes/perceptions/beliefs=2; Knowledge/awareness=3; Behavioural intentions/motivations=NR] |
| 25) What was the length of participant follow-up? | __________  Not reported  N/a – qualitative study | Specify the UNIT OF MEASUREMENT (e.g. hours, days, weeks, months, years) |
| 26) How were food safety outcome data measured/ collected? | Questionnaire:   - In-person - Phone - Postal - Web-based   Qualitative interviews:   - In-person - Phone - Web-based   Focus groups:   - In-person - Phone - Web-based   Participant observation, specify details ____  Analysis of documents, specify details ____  Other, please specify ______  Not specified |  |
| 27) Was the outcome measurement instrument assessed for any measurement properties? | Yes, specify:   - Validity - Reliability/reproducibility - Internal consistency - Other, please specify ______   None specified  N/a – qualitative study | **Only answer yes to any options if EXPLICITLY indicated by authors.**  **Validity:** Includes *content* (extent that all domains are covered), *face*/*construct* (instrument measures what it is intended to measure), and *criterion* validity (how well one variable or set of variables predicts an outcome based on information from other variables).  **Reliability/reproducibility:** Does the instrument measure the constructs in a reproducible fashion? (e.g. intra/inter-observer, test-retest).  **Internal consistency:** Extent to which items in a (sub)scale are intercorrelated, thus measuring the same construct (Cronbach’s alpha).  **Other:** could include *floor/ceiling effects* (many participants achieved highest or lowest scores), *interpretatability* (whether qualitative meaning can be assigned to quantitative scores), and *responsiveness* (is the instrument able to detect an important change due to the intervention?) |
| 28) Were intervention efficacy outcomes (relevant to food safety education) sufficiently reported to allow for possible meta-analysis? | Yes, all outcomes sufficiently reported, specify:   - Dichotomous - Continuous - Ordinal/Likert scale - Other:________________   Yes, some outcomes sufficiently reported, specify:   - Dichotomous - Continuous - Ordinal/Likert scale - Other:________________   No, all outcome insufficiently reported, specify reason:_________  N/A – qualitative research or intervention efficacy not reported | **Only answer this question with respect to INTERVENTION EFFICACY outcomes relevant to the review question – not other outcomes (e.g. participant characteristics, test validity/reliability measures).**  **Only answer based on how outcome data are REPORTED – not how measured in questionnaires/other instruments.**  **Dichotomous:** Sufficient information includes:   - - Numerator ***and*** denominator, ***or***   - proportion + EITHER numerator or denominator ***or*** - Measure of association (e.g. odds ratio, relative risk) + EITHER a measure of variability (SE, CIs, variance) *or* an exact P-value   **Continuous:** Sufficient information includes:   - - Mean, sample size, + EITHER a measure of variability (e.g. SD, CIs) *or* exact P-value/t-value ***or***   - Sample size and P-value/t-value from t-test ***or***   - Difference in means and a measure of variability (SD, SE, CIs, variance) ***or***   - Difference in means, sample size, + EITHER a common SD *or* an exact P-value /t-value   **Ordinal:** proportions reported for >2 ordinal/Likert scale categories (see required information for dichotomous data above). |
| 29) Additional comments: | __________ |  |

Risk-of-Bias Form

*For all risk-of-bias questions below, please provide justification for each response in the text boxes provided (e.g. by quoting relevant sections of the article)*

| **Screening questions** | **Response** | **Definitions/additional notes** |
| --- | --- | --- |
| Was the study conducted in a region of interest? | Yes  No | Relevant regions include North America, Europe and Australia/New Zealand.  ***If YES – proceed with remainder of the form***  ***If NO – submit form without proceeding further*** |
| Did the study include a control/ comparison group? | Yes  No | Includes pre-post comparisons (i.e. uncontrolled before-after studies).  ***If YES – proceed with remainder of the form***  ***If NO – submit form without proceeding further*** |
| Were any intervention efficacy outcomes sufficiently reported for potential use in meta-analysis? | Yes  No | ***If YES – proceed with remainder of the form***  ***If NO – submit form without proceeding further*** |
| Please confirm the study design | RCT  Non-randomized trial  Controlled before-after study (CBA)  Uncontrolled before-after study | **Controlled trial:** an experimental study in which people are allocated to intervention/comparison groups and evaluated for outcomes. Randomized (RCT) if authors specifically indicate random allocation of treatment/control.  **Controlled before-and-after (CBA) study:** A study in which observations are made before and after the implementation of an intervention, both in a group that receives the intervention and in a control group that does not.  **Uncontrolled before-and-after study:** observations are made on a single population (one group only) before and after receiving an intervention. |
| **Bias domain/ question** | **Risk of bias** | **Definitions/additional notes** |
| **Random sequence generation**  *Was the allocation sequence adequately generated?* | - Low - High - Unclear - N/A (non-randomized trials/uncontrolled before-after study) | Describe the method used to generate the allocation sequence (for allocating individuals into treatment groups) in sufficient detail to allow an assessment of whether it should produce comparable groups.  **Low:** a random component in the sequence generation process is described (e.g. referring to a random number table or computer random number generator)  **High:** a non-random method is used (e.g. performed by date of birth, by preference, or convenience).  **Unclear:** insufficient information provided to permit judgement. |
| **Allocation concealment**  *Was the allocation sequence adequately concealed from the participants and the researcher?* | - Low - High - Unclear - N/A (uncontrolled before-after study) | Describe the method used to conceal the allocation sequence in sufficient detail to determine whether intervention allocations could have been foreseen in advance of, or during, enrolment.  **Low:** the unit of allocation was by institution, team or professional and allocation was performed on all units at the start of the study; or if the unit of allocation was by participant, there was some form of centralised randomisation scheme such as an on-site computer system or sealed opaque envelopes were used.  **High:** a non-random method is used (e.g. performed by date of birth, by preference, or convenience).  **Unclear:** insufficient information provided to permit judgement. |
| **Similarity of baseline outcomes**  *Were baseline measurements taken and results compared across groups to ensure comparability?* | - Low - High - Unclear - N/A (uncontrolled before-after study) | Note whether baseline outcome measurements were reported and whether there were any important differences between groups. If there were important differences between groups, note whether appropriate adjusted analysis was performed to account for this.  **Low:** participant outcomes were measured prior to the intervention, and no important differences were present across study groups. Score “Low” if imbalanced but appropriate adjusted analysis was performed (e.g. analysis of covariance/regression).  **High:** important differences were present and not adjusted for in analysis.  **Unclear:** If trials have no baseline measure of outcome. |
| **Similarity of baseline characteristics**  *Were participant demographics compared across groups to ensure comparability?* | - Low - High - Unclear - N/A (uncontrolled before-after study) | Note whether baseline characteristics were reported and whether there were any important differences between groups.  **Low:** baseline characteristics of the study and control participants are reported and similar.  **High:** there is no report of characteristics in text or tables or there are important differences between control and intervention participants and these were not controlled/accounted for in the analysis and results.  **Unclear:** it is not clear in the paper (e.g. characteristics are mentioned in text but no data were presented). |
| **Blinding of participants and personnel**  *Were participants and personnel blinded from knowledge of intervention/ control group status?* | - Low - High - Unclear - N/A (uncontrolled before-after study) | Describe all measures used, if any, to blind study participants and personnel from knowledge of which intervention a participant received. Provide any information relating to whether the intended blinding was effective.  **Low:** no blinding of outcome assessment, but the review authors judge that the outcome measurement is not likely to be influenced by lack of blinding. Blinding of outcome assessment ensured, and unlikely that the blinding could have been broken.  **High:** no blinding of outcome assessment, and the outcome measurement is likely to be influenced by lack of blinding. Blinding reported, but likely that the blinding could have been broken, and the outcome measurement is likely to be influenced by lack of blinding.  **Unclear:** insufficient information provided to permit judgement. |
| **Blinding of participants to the research question/purpose**  *Were participants blinded from knowledge of the research question/ purpose?* | - Low - High - Unclear - N/A (controlled trials) | Describe all measures used, if any, to blind study participants from the research question/purpose  **Low:** the authors state explicitly that participants were blinded to the research question  **High:** participants not blinded.  **Unclear:** insufficient information provided to permit judgement. |
| **Blinding of outcome assessment**  *Were outcome assessors blinded from knowledge of which intervention a participant received?* | - Low - High - Unclear - Separate risk for different outcomes, specify: - N/A (uncontrolled before-after study) | Describe all measures used, if any, to blind outcome assessors from knowledge of which intervention a participant received.  **Low:** the authors state explicitly that the primary outcome variables were assessed blindly, or outcome is objective (e.g. microbial counts).  **High:** outcomes were not assessed blindly (e.g. participants self-reported their own outcomes and were not blinded to intervention/ control group status).  **Unclear:** insufficient information provided to permit judgement.  ***Assessments should be made for each main outcome (or class of outcomes), as appropriate.*** *If more than one answer please specify which outcomes are associated with each answer.* |
| **Independence of intervention effect from confounding bias**  *Are there any concerns that confounders have not been appropriately identified and accounted for?* | - Low - High - Unclear | Describe whether or not the intervention occurred independently of other changes over time and whether or not the outcomes may have been influenced by other confounding variables/historic events during the study period.  *Important confounders could include***:** age; sex; race/ethnicity; marital/family status; socio-economic status (income/class); education/literacy levels; health/immune status.  **Low:** there are compelling arguments that the intervention occurred independently of other changes over time and the outcome was not influenced by other confounding variables and/or historic events during study period. If randomization conducted (e.g. RCT), indicate LOW.  **High:** intervention was likely not independent of other changes in time.  **Unclear:** insufficient information provided to permit judgement. |
| **Valid/reliable outcome measurement**  *Were outcome measurement instruments valid and reliable?* | - Low - High - Unclear - Separate risk for different outcomes, specify: | Describe whether the method(s) of outcome assessment could have influenced or biased the results.  **Low:** authors report using validated (e.g. pre-tested) measurement instruments and reliability tested outcome scales/scores.  **High:** use of non-validated instruments and/or non-reliability tested outcome scores/scales.  **Unclear:** not specified in the paper.  ***Assessments should be made for each main outcome (or class of outcomes), as appropriate.*** *If more than one answer please specify which outcomes are associated with each answer.* |
| **Incomplete outcome data**  *Were losses to follow-up (attrition) and exclusions from analysis reported and comparable in both groups?* | - Low - High - Unclear - Separate risk for different outcomes, specify: | Describe the completeness of outcome data for each main outcome, including attrition and exclusions from the analysis. State whether attrition and exclusions were reported, the numbers in each intervention group (compared with total randomized participants), reasons for attrition/exclusions where reported, and any re-inclusions in analyses performed by the review authors.  **Low:** missing outcome measures were unlikely to bias the results (e.g. the proportion of missing data was similar in the intervention and control groups or the proportion of missing data was less than the effect size - i.e. unlikely to overturn the study result).  **High:** missing outcome data likely to bias the results.  **Unclear:** not specified in the paper (Do not assume 100% follow up unless stated explicitly).  ***Assessments should be made for each main outcome (or class of outcomes), as appropriate.*** *If more than one answer please specify which outcomes are associated with each answer.* |
| **Selective reporting**  *Did the authors report all intended outcomes?* | - Low - High - Unclear | State how the possibility of selective outcome reporting was examined by the review authors, and what was found.  **Low:** there is no evidence that outcomes were selectively reported (e.g. all relevant outcomes in the methods section are reported in the results section).  **High:** some important outcomes are omitted from the results.  **Unclear:** insufficient information provided to permit judgement. |
| **Other**  *Was the study free of other problems that could put it at a high risk of bias?* | - Low - High - Unclear | State any important concerns about bias not addressed in the other domains in the tool (e.g. study funded by industry with concerns about sponsor involvement, inadequate control of hierarchical data structure, or possible contamination/exposure of control group to the intervention)  **Low:** there is no risk of other biases (please specify details).  **High:** there is a risk of other biases (please specify details).  **Unclear:** possible risk of other biases but insufficient information provided to permit judgement (please specify details). |
| **Overall risk-of-bias for each outcome (within-study summary assessment)** | - Low - High - Unclear - Separate risk for different outcomes, specify: | **Low:** plausible bias unlikely to seriously alter the results. Low risk of bias for key domains.  **High:** plausible bias that seriously weakens confidence in the results. High risk of bias for key domains.  **Unclear:** plausible bias that raises some doubt about the results. Unclear risk of bias for key domains.  ***Assessments should be made for each main outcome (or class of outcomes), as appropriate.*** *If more than one answer please specify which outcomes are associated with each answer.* |

Data Extraction Form

*Multiple forms should be submitted for each unique trial (i.e. intervention/population/outcome combination) reported in a study*

| **Question** | **Options** |
| --- | --- |
| Specify intervention category and copy and paste the full intervention description/details from the paper into the corresponding text box. | Training workshop/session:______________  Preschool to high school course/curriculum:______________  University/college course/curriculum:______________  Media campaign/social marketing/other messaging:________ |
| Specify target population category and copy and paste any key inclusion criteria/characteristics in the corresponding text box | Consumers:______________  Educators of consumers:______________ |
| Specify outcome category and copy and paste the full outcome description/details in the corresponding text box | Attitudes/perceptions/beliefs:______________  Knowledge/awareness:______________  Behaviours:______________  Microbial prevalence/counts :______________  Incidence of foodborne illness:______________  Overall/combined score or scale (combining multiple measures of the above, specify):______________  Other, specify:______________ |
| Specify the outcome measurement instrument and outcome scale copy and paste details in the corresponding text box | Questionnaire:____________  Observation score:______________  Counts of microbes/illness:______________  Other:______________ |
| Does this trial include indirect measurement of the intervention, population, comparison, and/or outcome of interest? | Yes  No  **A study may indirectly address the question of interest if:**  *E.g. interventions we wish to compare are measured independently in two separate trials compared to controls.*  *E.g. the population, intervention, comparisons or outcomes were not exactly what we are trying to draw conclusions for. (E.g., surrogate outcomes = self-reported vs. actual/observed behaviours; OR food safety outcomes collapsed as part of overall score/scale with other measures such as nutrition).* |
| Is there reason to believe that due to the population studied, the magnitude of effect of the intervention is likely to be underestimated? | Yes  No  ***ONLY answer yes* *if there is good reason to think that the study underestimated the potential association or effect of an intervention due to the population that was sampled. (I.e. all plausible biases working to underestimate apparent intervention effect).***  *E.g. intervention was tested only on individuals with prior food safety knowledge/training, and it is likely that a better success rate would have been found if the intervention was tested in ALL individuals (e.g. including those with no prior knowledge/training).* |
| Was a dose-response gradient detected for the intervention effect? | Yes  No  **If a dose response gradient is demonstrated in some or all of the studies, this increases our confidence in the findings of the study and thus we can consider upgrading the evidence.** |
| Specify outcome data format  *NOTE: For RCTs, only extract the FINAL outcomes across each intervention/control group, not the pre-post comparisons within each group (if reported). CBA studies could report matched (pre-post within groups, difference in change from baseline) and/or unmatched data (comparison of final outcomes), please extract both.* | □ Dichotomous data  □ Unmatched (RCTs, CBAs, controlled trials)  □ Matched (matched - uncontrolled before-after studies/CBAs/cross-over trials)  □ Continuous data  □ Unmatched (RCTs, CBAs, controlled trials)  □ Matched (matched - uncontrolled before-after studies/CBAs/cross-over trials)  □ Ordinal data  □ Unmatched (RCTs, CBAs, controlled trials)  □ Matched (matched - uncontrolled before-after studies/CBAs/cross-over trials) |
| Were detailed questionnaire responses reported along with overall scores/scales? | Yes, specify details:  No |
| Extract quantitative outcome data in text boxes for each relevant category | **Dichotomous/ordinal data options**  □ Raw 2X2 data  □ Number positive group 1  □ Number negative group 1  □ Number positive group 2  □ Number negative group 2  □ Define group 1  □ Define group 2  □ Specify “positive”  □ Specify “negative”  □ For matched studies, indicate external correlation  □ For ordinal data, specify above for additional response categories as appropriate:  □ Computed effect size / measure of association (e.g. OR):  □ Measure of association value  □ Specify measure (e.g. OR, RR, etc.)  □ N in group 1  □ N in group 2  □ Define group 1  □ Define group 2  □ SE  □ Variance  □ Lower CI  □ Higher CI  □ Was the outcome adjusted for other variables? If yes, check this box and specify:______  **Continuous data options**  □ Raw continuous data in each group (final outcome measure):  □ Counts in group 1  □ SD in group 1  □ N in group 1  □ Counts in group 2  □ SD in group 2  □ N in group 2  □ Define group 1  □ Define group 2  □ P-value *(exact Ps only)*  □ T-value  □ For matched studies, indicate pre/post correlation  □ Outcome units  □ Outcome scale (i.e. lowest and highest possible values)  □ Raw pre-post data in one or more groups  □ Counts in group 1 - pre  □ SD in group 1 - pre  □ N in group 1 – pre  □ Counts in group 1 - post  □ SD in group 1 - post  □ N in group 1 – post  □ Define group 1  □ P-value *(exact Ps only)*  □ T-value  □ For matched studies, indicate pre/post correlation  □ Outcome units  □ Outcome scale (i.e. lowest and highest possible values)  □ Counts in group 2 - pre  □ SD in group 2 - pre  □ N in group 2 – pre  □ Counts in group 2 - post  □ SD in group 2 - post  □ N in group 2 – post  □ Define group 2  □ P-value *(exact Ps only)*  □ T-value  □ For matched studies, indicate pre/post correlation  □ Outcome units  □ Outcome scale (i.e. lowest and highest possible values)  □ Difference in means (between intervention/control groups):  □ Difference in means (value)  □ N (total sample size)  □ Common SD  □ SE  □ Variance  □ Lower CI  □ Higher CI  □ P-value *(exact Ps only)*  □ T-value  □ Outcome units  □ Outcome scale (i.e. lowest and highest possible values)  □ Was the outcome adjusted for other variables? If yes, check this box and specify:______  □ Mean change from baseline (between pre-post values):  □ Specify group  □ Difference in means (value)  □ N (total sample size)  □ Common SD  □ SE  □ Variance  □ Lower CI  □ Higher CI  □ P-value *(exact Ps only)*  □ T-value  □ Outcome units  □ Outcome scale (i.e. lowest and highest possible values)  □ Was the outcome adjusted for other variables? If yes, check this box and specify:______  □ Other:__________________ |
