# Supplementary material for: A systematic review and meta-analysis of the effectiveness of food safety education interventions for consumers in developed countries
Source: BMC Public Health. 2015 Aug 26;15:822. doi: 10.1186/s12889-015-2171-x (PMC4548310; doi:10.1186/s12889-015-2171-x)
Supplement: Additional file 5: — List of studies with more than two intervention and control groups. (DOCX 25 kb) [file 12889_2015_2171_MOESM5_ESM.docx]

Additional File 5: List of Studies with More than Two Intervention/Control Groups

| **Reference** | **Original compared groups** | **Combined groups for meta-analysis** |
| --- | --- | --- |
| Gold et al., 2014 | Cooking class  Map class  Control | Intervention = cooking class + map class  Control = control |
| Bearth et al., 2013 | Informational brochure and pictorial postcard  Informational brochure and colored cutting boards  Control | Intervention = both informational brochure groups  Control = control |
| Chipman et al., 1996 | Video news release  Video public service announcement  Print news release  Newsprint column | Intervention = video release + video announcement  Control = print release + news column |
| Haapala 2001 | Computer-mediated cooperative learning assignment  Face-to-face cooperative assignment  Control | Intervention 1 = computer-mediated learning  Control 1 = control (half sample size)  Intervention 2 = face-to-face learning  Control 2 = control (half sample size) |
| Hovis et al., 2007 | Internet-based lesson  Standard lesson  Modified standard lesson | Intervention = Internet lesson  Control = standard lessons |
| Mayer and Harrison, 2012 | Facebook page access (≥15 min/week)  Facebook page access (<15 min/week)  Lecture only  Lecture + Facebook page access (≥15 min/week)  Lecture + Facebook page access (<15 min/week)  Control | Intervention 1 = Facebook only  Control 1 = control (one third sample size)  Intervention 2 = Lecture only  Control 2 = control (one third sample size)  Intervention 3 = Lecture + Facebook  Control 3 = control (one third sample size) |
| Verbeke et al., 2008 | Benefit-only message  Risk-only message  Balanced benefit-risk message  Balanced risk-benefit message | Intervention = risk-only message  Control = other messages |
