# Supplementary material for: A systematic review and meta-analysis of the effectiveness of food safety education interventions for consumers in developed countries
Source: BMC Public Health. 2015 Aug 26;15:822. doi: 10.1186/s12889-015-2171-x (PMC4548310; doi:10.1186/s12889-015-2171-x)

Additional File 9: Forest Plots for Each Meta-Analysis Subgroup

NOTE: For all forest plots below, the average estimate of effect (represented by a diamond at the bottom of the figure) is shown only if heterogeneity was not significant (*I*^2^<60%)

*Randomized controlled trials (RCTs)*

Training courses/workshops – adults – behaviour outcome – standardized mean difference (SMD) measure


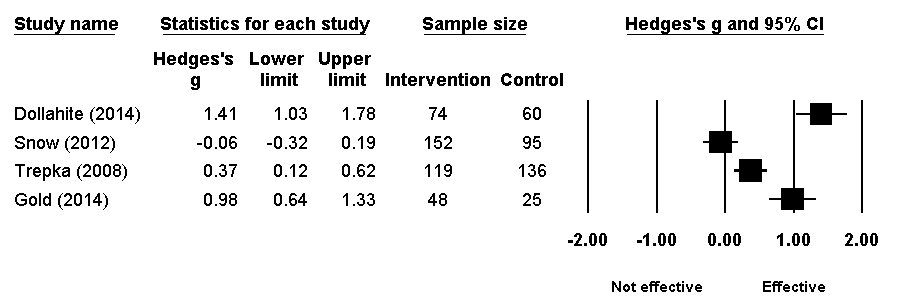


Training courses/workshops – adults – knowledge outcome – SMD measure


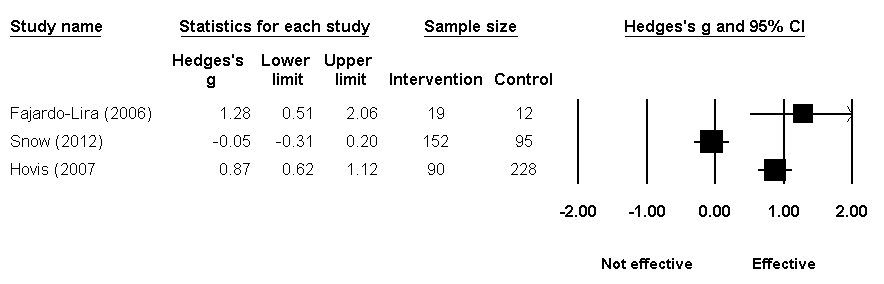


Training courses/workshops – children/youth – behaviour outcome –SMD measure


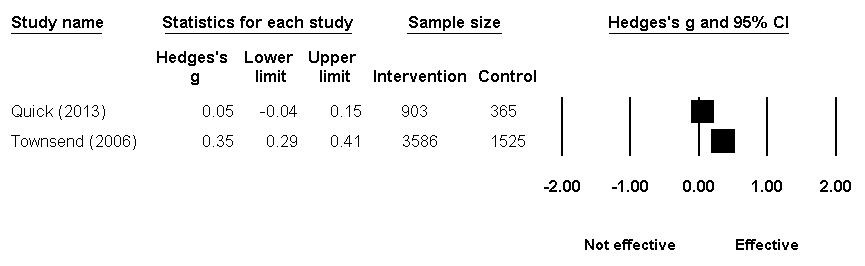


Media campaigns/messaging – adults – behavioural intentions outcome – SMD measure


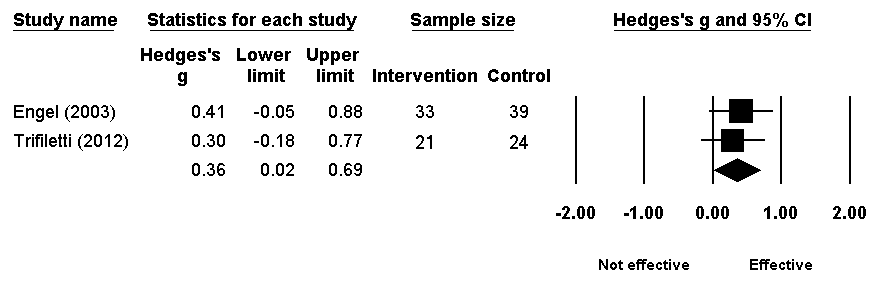


Media campaigns/messaging – adults – behaviour outcome – SMD measure


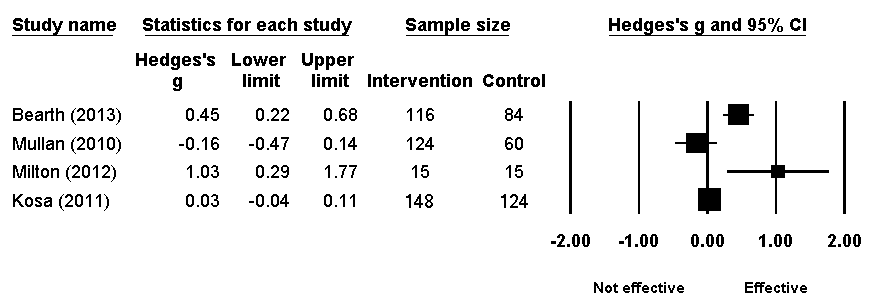


Media campaigns/messaging – adults – knowledge outcome – SMD measure


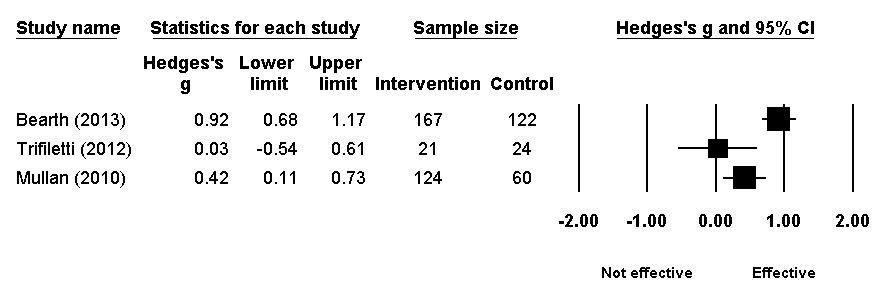


Media campaigns/messaging – adults – attitudes outcome – SMD measure


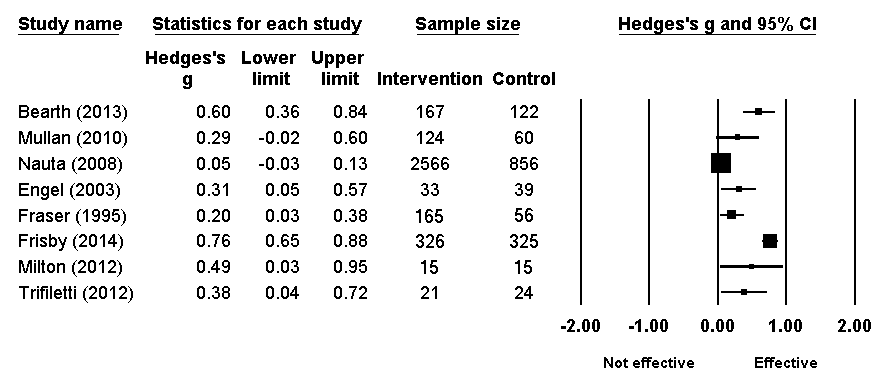


*Non-randomized controlled trials (NRTs)*

Training courses/workshops – adults – behaviour outcome –SMD measure


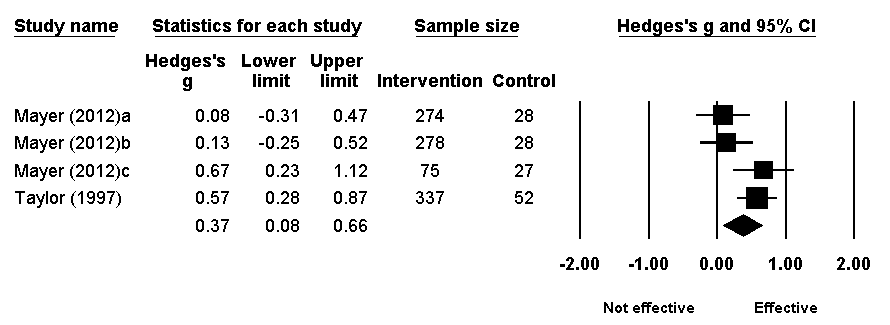


Training courses/workshops – adults – knowledge outcome –SMD measure


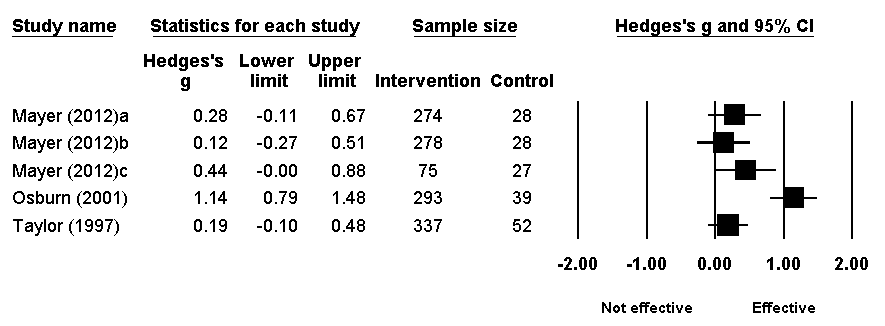


Training courses/workshops – adults – attitudes outcome –SMD measure


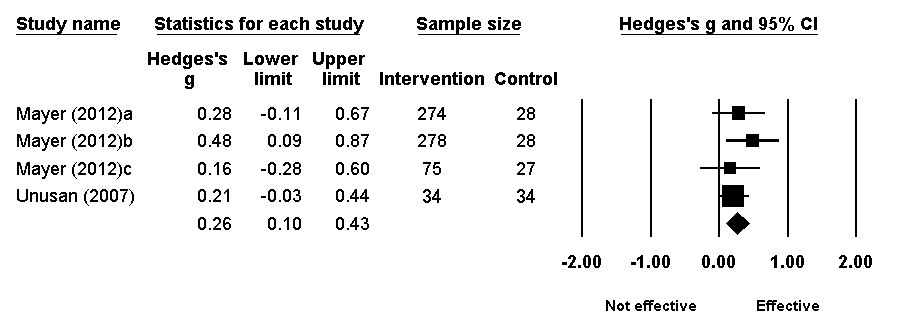


Training courses/workshops – children/youth – behaviour outcome –SMD measure


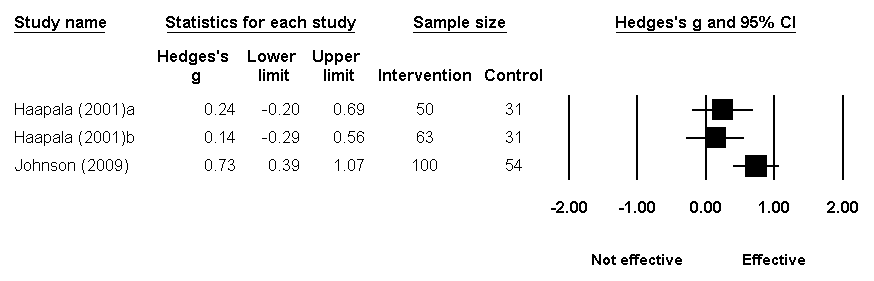


Training courses/workshops – children/youth – knowledge outcome –SMD measure


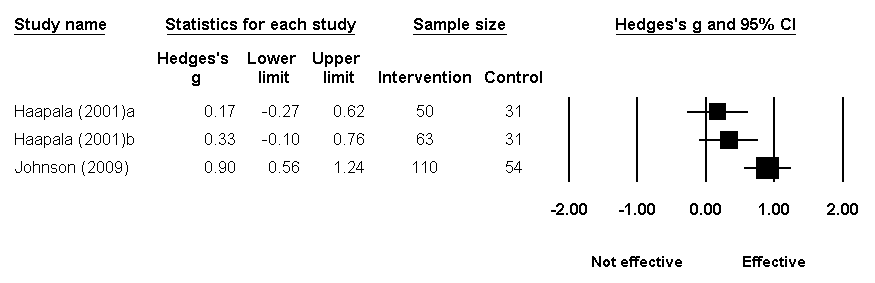


Media campaigns/messaging – adults – behaviour outcome – relative risk (RR) measure (shown on log scale)


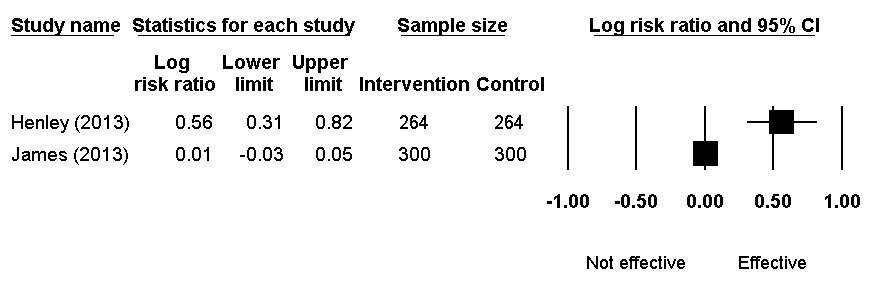


Media campaigns/messaging – adults – attitudes outcome – RR measure (shown on log scale)


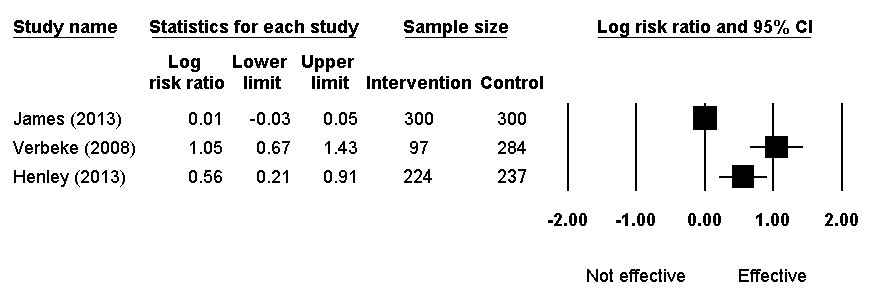


*Uncontrolled before-and-after studies*

Training courses/workshops – educators – behaviour outcome – SMD measure


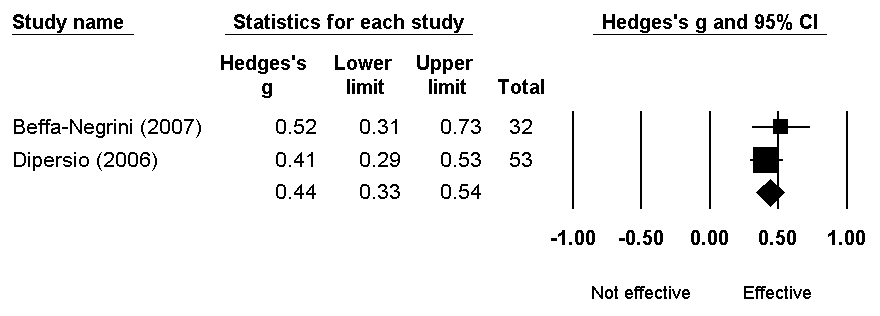


Training courses/workshops – educators – knowledge outcome – RR measure (shown on log scale)


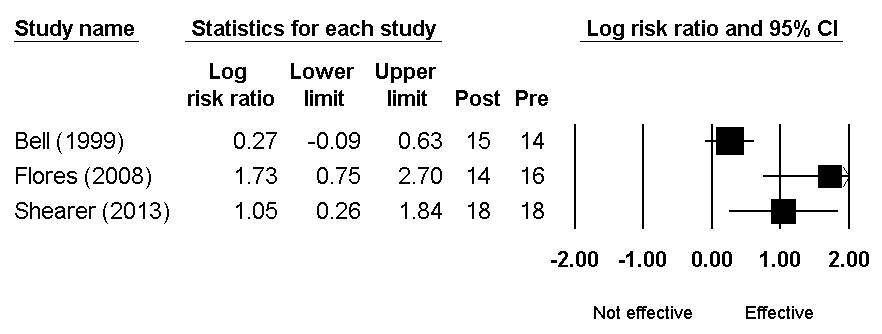


Training courses/workshops – educators – attitudes outcome – RR measure (shown on log scale)


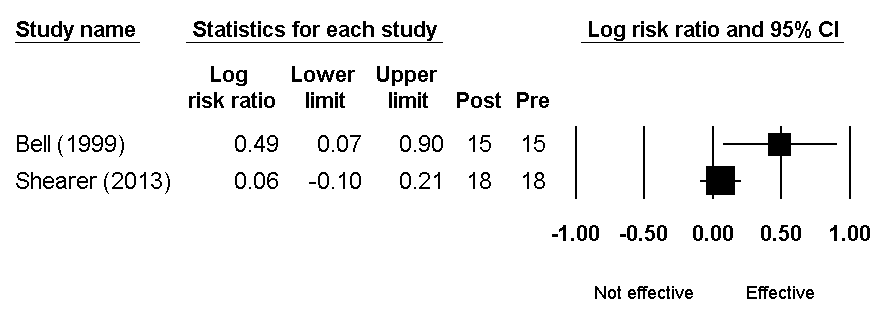


Training courses/workshops – adults – behaviour outcome – SMD measure


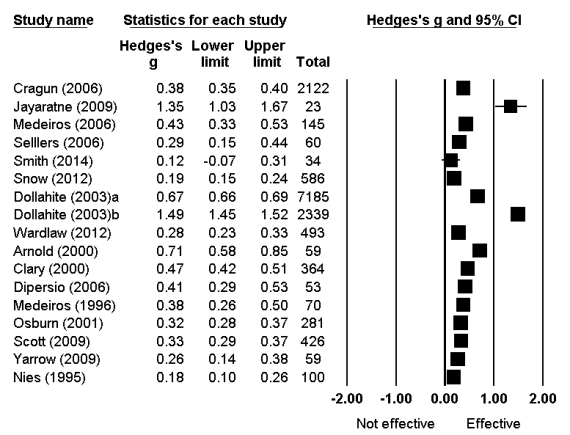


Training courses/workshops – adults – behaviour outcome – RR measure (shown on log scale)


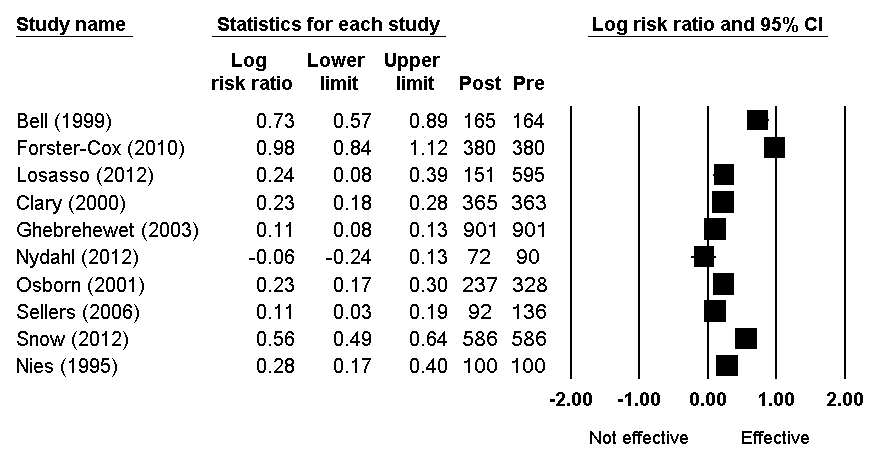


Training courses/workshops – adults – knowledge outcome – SMD measure


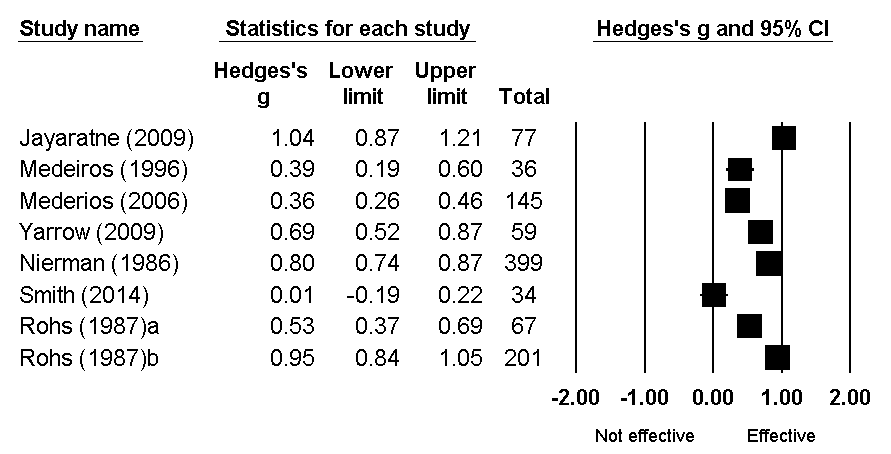


Training courses/workshops – adults – knowledge outcome – RR measure (shown on log scale)


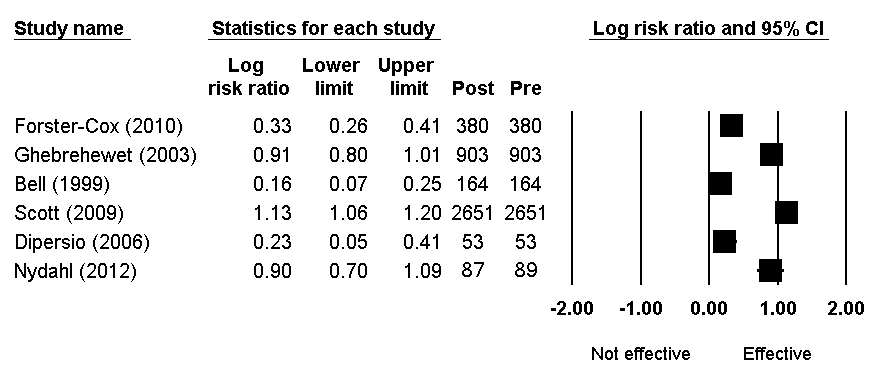


Training courses/workshops – adults – attitudes outcome – SMD measure


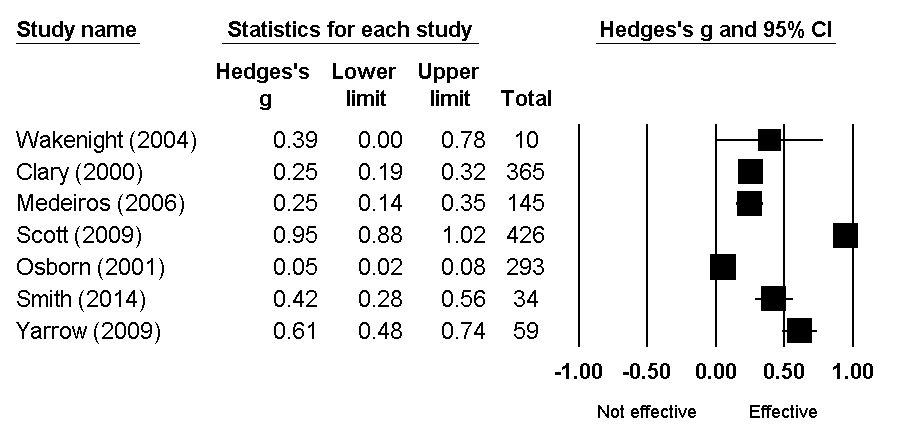


Training courses/workshops – adults – attitudes outcome – RR measure (shown on log scale)


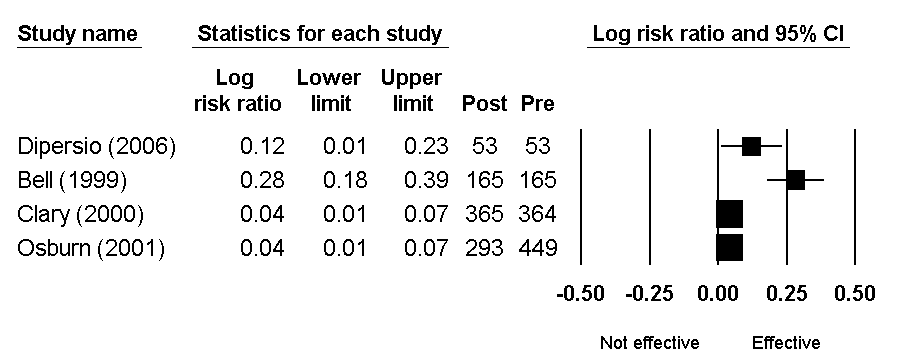


Training courses/workshops – children/youth – behaviour outcome – SMD measure


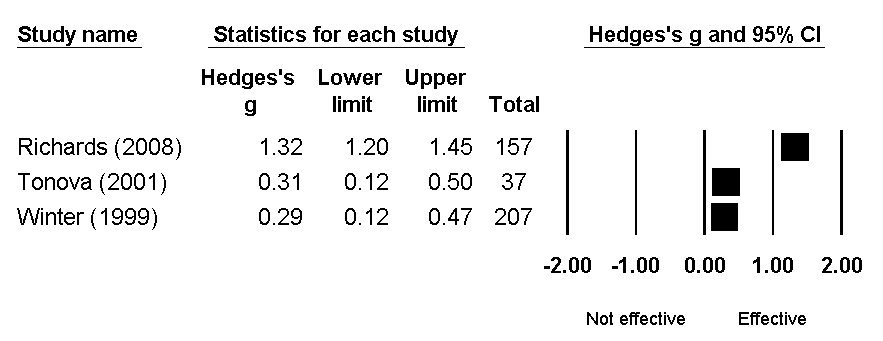


Training courses/workshops – children/youth – behaviour outcome – RR measure (shown on log scale)


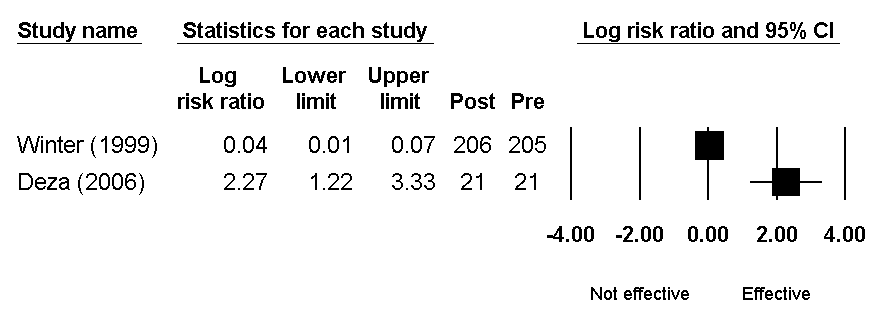


Training courses/workshops – children/youth – knowledge outcome – SMD measure


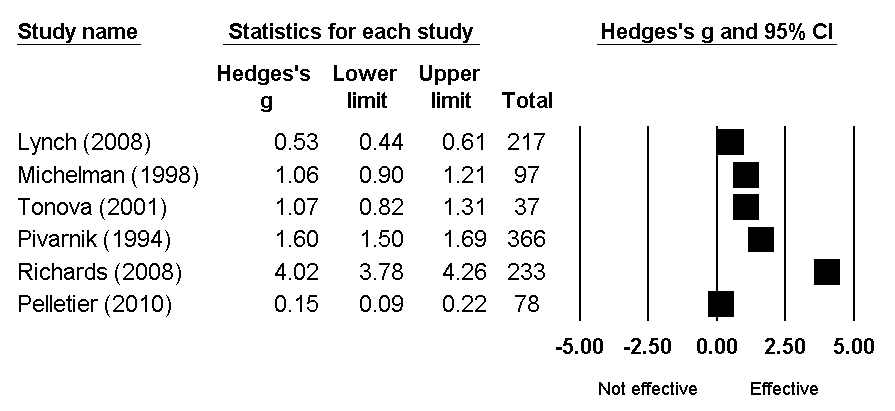


Training courses/workshops – children/youth – knowledge outcome – RR measure (shown on log scale)


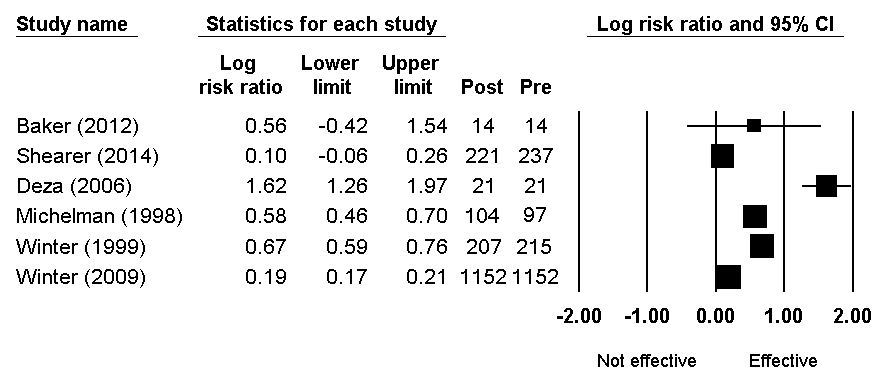


Training courses/workshops – children/youth – attitudes outcome – SMD measure


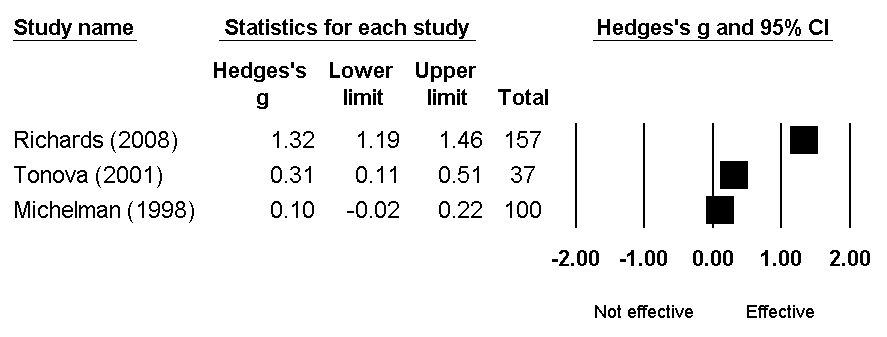


Media campaigns/messaging – adults – behaviour outcome – RR measure (shown on log scale)


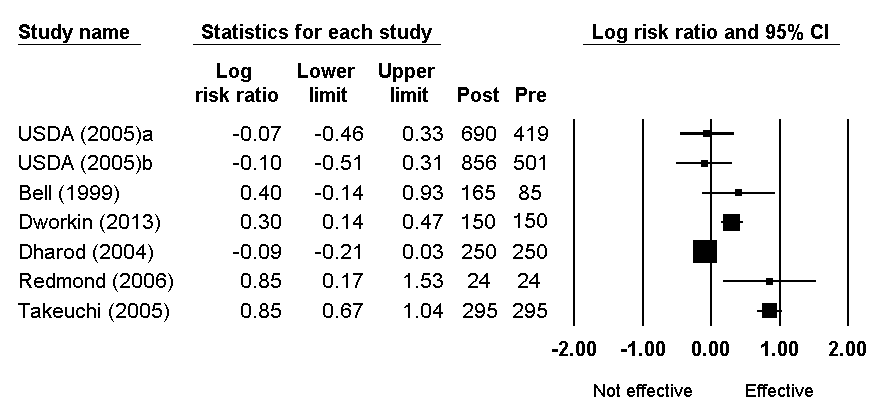


Media campaigns/messaging – adults – knowledge outcome – RR measure (shown on log scale)


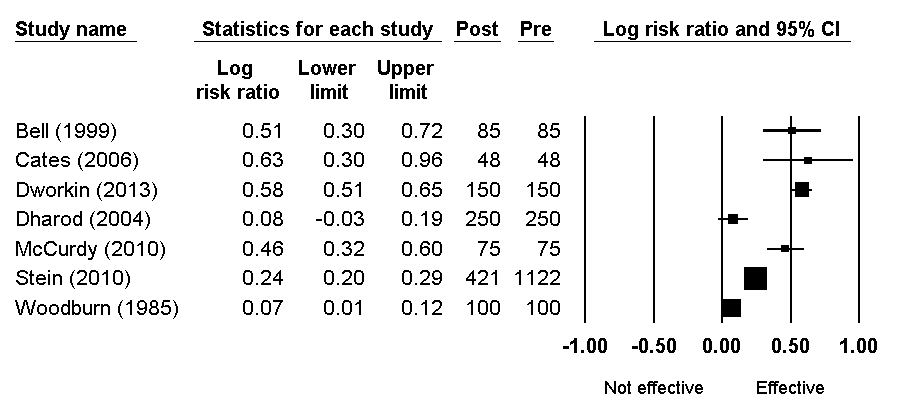


Media campaigns/messaging – adults – attitudes outcome – SMD measure


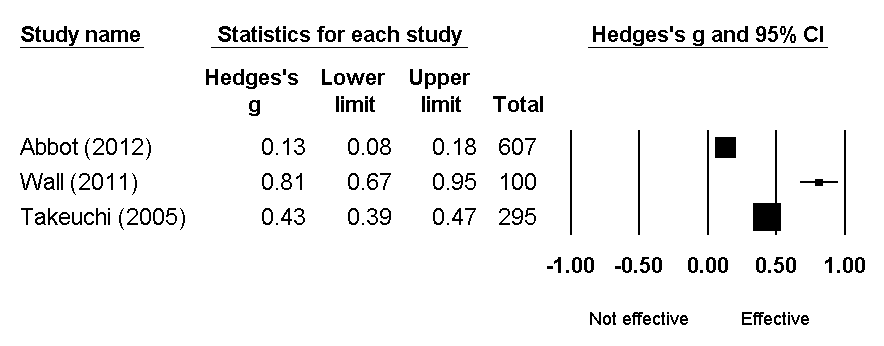


Media campaigns/messaging – adults – attitudes outcome – RR measure (shown on log scale)


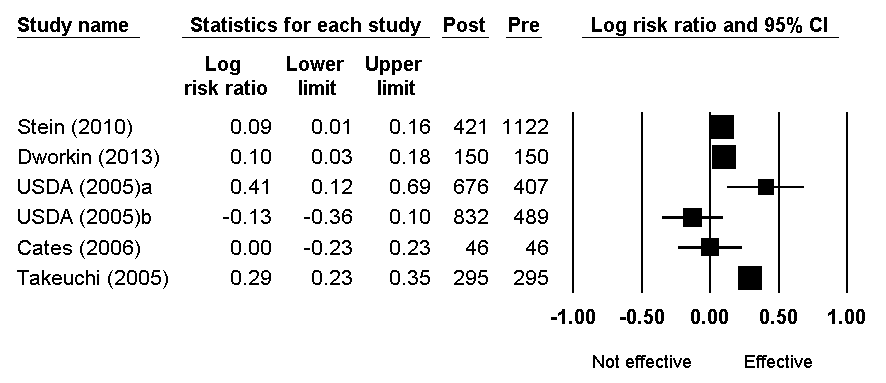


Media campaigns/messaging – adults – stages of change outcome – RR measure (shown on log scale)


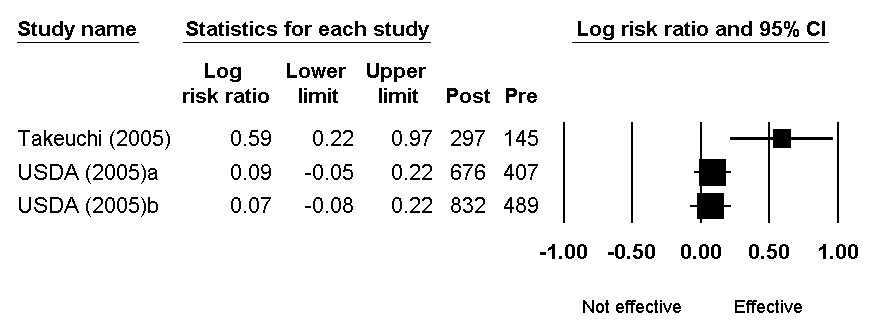

Supplement: Additional file 9: — Forest plots for each meta-analysis subgroup. (DOCX 527 kb) [file 12889_2015_2171_MOESM9_ESM.docx]
